# Boron Nitride Nanosheet–Magnetic Nanoparticle Composites for Water Remediation Applications

**DOI:** 10.1021/acsomega.3c06593

**Published:** 2024-01-15

**Authors:** Garret Dee, Olivia O’Donoghue, Eoin Devitt, Tiphaine Giroud, Aran Rafferty, Lee Gannon, Cormac McGuinness, Yurii K. Gun’ko

**Affiliations:** † School of Chemistry, University of Dublin, Trinity College, Dublin 2, Ireland; ‡ SIGMA Clermont, Campus De Clermont-Ferrand, 63178 Aubiere Cedex, France; § School of Physics University of Dublin, Trinity College, Dublin 2, Ireland

## Abstract

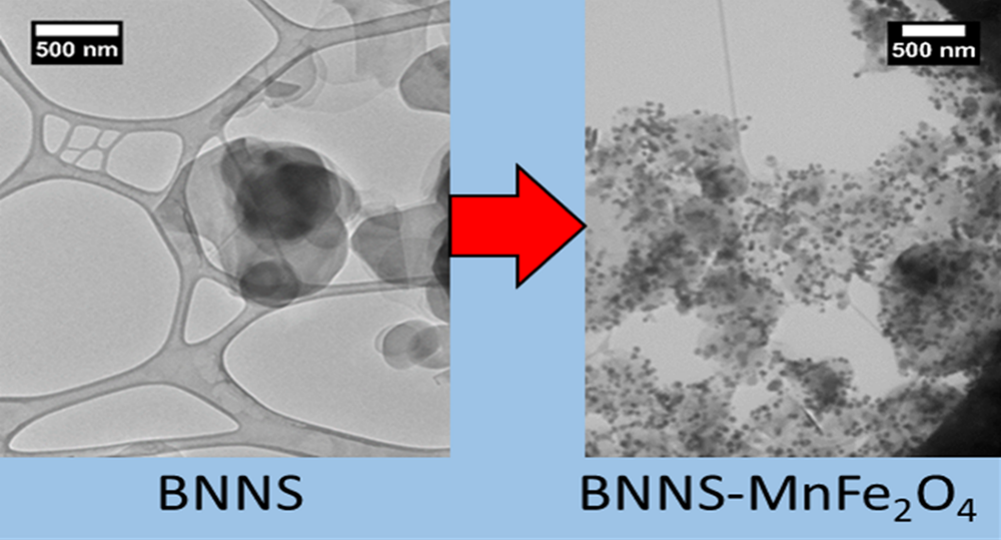

The combination of 0D nanoparticles with 2D nanomaterials has attracted a lot of attention over the last years due to the unique multimodal properties of resulting 0D-2D nanocomposites. In this work, we developed boron nitride nanosheets (BNNS) functionalized with manganese ferrite magnetic nanoparticles (MNPs). The functionalization process involved attachment of MNPs to exfoliated BNNS by refluxing the precursor materials in a polyol medium. Characterization of the produced BNNS-MNP composites was carried out using powder X-ray diffraction, transmission electron microscopy, vibrating sample magnetometry, Fourier transform infrared spectroscopy, and X-ray photoelectron spectroscopy. The adhesion of MnFe_2_O_4_ magnetic nanoparticles onto the BNNS remained unaffected by repeated sonication and heating in a furnace at 400 °C, underscoring the robust nature of the formed bond. FTIR spectra and XPS deconvolution confirmed the presence of strong bonding between BNNS and the MNPs. Membranes were fabricated from the BNNS and the BNNS-MnFe_2_O_4_ nanocomposites for evaluating their efficiency in removing the methylene blue dye pollutant. The membranes have been characterized by scanning electron microscopy, Brunauer–Emmett–Teller surface area analysis, and mercury intrusion porosimetry. The effectiveness of dye removal was monitored using ultraviolet–visible spectroscopy. The BNNS-MnFe_2_O_4_ nanocomposite membranes exhibited enhanced MB capture compared to membranes made from pure BNNS alone. The recyclability assessment of BNNS-MnFe_2_O_4_ demonstrated exceptional performance, retaining 92% efficiency even after eight cycles. These results clearly demonstrate the high potential of these magnetic nanocomposites as reusable materials for water filtration membranes. Furthermore, the introduction of magnetic functionality as part of the membrane brings an exciting opportunity for in situ magnetic heating of the membrane, which shall be explored in future work.

## 1 Introduction

In the modern world, environmental pollution poses a significant and pressing challenge, encompassing various forms, such as water pollution. Numerous man-made chemicals exhibit remarkable resistance to breakdown in the environment by natural means, leading to their role as environmental pollutants. This category includes pesticides, herbicides, pharmaceuticals, oils, polycyclic aromatic hydrocarbons, and artificial dyes.^1^ Among these, synthetic dyes find extensive usage across diverse industries such as paper, plastic, leather, and textiles. A majority of synthetic dyes possess inherent toxicity and show formidable resistance to degradation due to their intricate molecular structures. Consequently, they are classified as hazardous organic compounds in the environment, with methylene blue exposure having been reported to cause unwanted symptoms.^2^ The conspicuous and undesirable visibility of even trace amounts of these dyes in water accentuates the issue. Thus, the appropriate disposal of synthetic dyes remains a subject of environmental concern. Removal of these dyes from the environment is critical with many research technologies having this as a main goal.^3^ Adsorption of pollutants onto suitable sorbents is an efficient route to remove these pollutants from the environment with boron nitride being a potentially promising candidate.^4^

Boron nitride (BN) is a material with several crystalline polymorphs such as the cubic, wurtzite, and hexagonal (h-BN) phases. h-BN has the moniker “white graphene” because of its similarity in structure with graphene, as it is composed of 2D layers of hexagonal rings of alternating B and N atoms, creating a honeycomb-type structure equivalent to graphene. Bulk h-BN can be exfoliated in water to produce individual 2D boron nitride nanosheets (BNNSs).^5^ BNNSs have unique properties such as high surface area and high thermal stability; they are chemically inert and stable to oxidation. These unique properties make the BNNS an attractive material for applications such as pollutant removal,^3^ lubricants,^6^ sensing applications,^7^ and super hydrophobic coatings.^8^ Magnetic nanoparticles are another material with unique properties, namely, their magnetic functionality and large surface area. Magnetic nanoparticles include a broad range of materials such as the spinel ferrite class (e.g., Fe_3_O_4_, MnFe_2_O_4_, and CoFe_2_O_4_). Magnetic nanoparticles and their composites have found application in targeted drug delivery,^9^ magnetic resonance imaging (MRI) diagnostics,^10^ magnetic heating in cancer hyperthermia therapy,^11^ and data storage.^12^ MNPs can be prepared by numerous methods, which include coprecipitation,^13^ thermal decomposition,^14^ and thermal synthesis.^15^

Boron nitride nanosheet-magnetic nanoparticle (BNNS-MNP) nanocomposites are a relatively new type of 2D-0D composite material with few papers published in this area. One of the reasons for this is because of the great challenge in attaching magnetic nanoparticles to the boron nitride sheets. Boron nitride, as mentioned above, is chemically inert and so is not amiable to functionalization. As a result of this, very harsh methods have previously been employed to add functional groups to the BNNS. One report involved reacting h-BN in the presence of di*tert*-butyl peroxide, which decomposes at 120 °C by homolytic fission to produce oxygen radicals, which then attack the BN sheets to produce *tert*-butyl functionalized BNNSs. Further reacting with piranha solution produced hydroxyl functionalized nanosheets (HO-BNNS).^16^ Another report involved the reacting of the BN with 5 M NaOH solution at 120 °C for 48 h. These harsh reaction conditions created hydroxyl functional groups on the BN.^17^ There is a report of attaching Fe_3_O_4_ to the surface of the BNNS with an in situ coprecipitation, but the TEM images in this article are inconclusive and unclear regarding the attachment and coverage of magnetic material.^18^ In another paper, an aerogel of BN with Fe_3_O_4_ has been reported.^19^ This aerogel showed the ability to remove both organic dyes and toxic metal ions from water, but again, the TEM images showed some MNPs to be separate from the BN material.

In this work, we have functionalized BNNSs with MnFe_2_O_4_ magnetic nanoparticles resulting in the BNNS coated with the MNPs with no separate MNPs in the samples. We demonstrated that high coverage of BNNS with MNPs, can be achieved through a process devoid of harsh chemical conditions. Our method is reproducible and effective in coating the BNNS with the spinel ferrite to make the BNNS-MNP nanocomposites. Then, the produced BNNS-MnFe_2_O_4_ nanocomposites were used to prepare new membranes, which were tested for nanofiltration applications. The membranes exhibited remarkable efficiency (over 99% retention before saturation) in eliminating MB dye pollutants from water, while also displaying the qualities of recyclability and sustained removal efficiency across multiple cycles. To the best of our knowledge, this composite material has not been reported in the literature to date. The added magnetic functionality brings with it the potential for inductive magnetic heating regeneration of the membrane to burn off and remove any adsorbed organic pollutant, an application which our group is currently exploring.

## 2 Results and Discussion

### 2.1 BNNS Preparation and Characterization

As mentioned earlier, h-BN is a layered material with a honeycomb-like structure, and in order to obtain individual nanosheets, it must first be exfoliated. Liquid-phase exfoliation was chosen for this work, as it is an easy and low-cost method of producing exfoliated 2D materials without the use of complex equipment or hazardous chemicals. We have followed the procedure, which has been previously reported by our group on BN exfoliation in water to produce BNNS.^5^ After the exfoliation of h-BN in water, a milky white suspension was obtained. The product was characterized by transmission electron microscopy and scanning electron microscopy (TEM and SEM), X-ray diffraction (XRD), Fourier transform infrared (FTIR) spectroscopy, and X-ray photoelectron spectroscopy (XPS).

**Figure 1:**
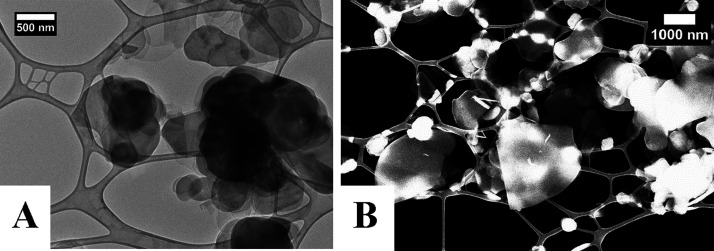
(A) TEM and (B) SEM images of BNNS on a Lacey carbon TEM grid.

TEM images were taken on Lacey carbon grids, with TEM and SEM images of a blank grid shown for reference (Figure S1). TEM and SEM images of the BNNS nanosheets on the Lacey carbon grids are shown (Figure 1), where we can see the exfoliated h-BN sample containing many thin layers of nanosheets with some aggregation occurring due to the drying process. The sheets vary from around 100–2000 nm in diameter. This large size distribution is common for liquid-phase exfoliation.^20^ Flake size distributions for the BNNS were calculated from 50 nanosheets in the TEM images using ImageJ software. A size distribution chart is shown in the Supporting Information (Figure S2).

**Figure 2:**
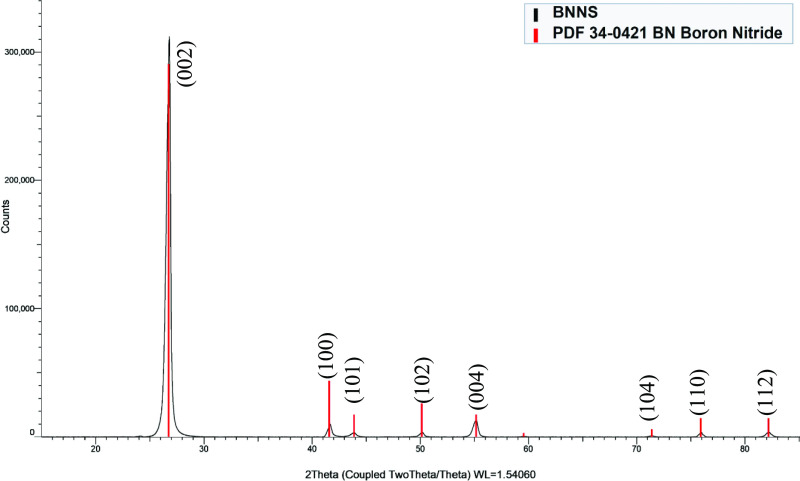
XRD pattern of exfoliated BNNS, showing the *hkl* planes for the major peaks.

The XRD pattern of the BNNS has the characteristic BN peaks at 2θ° with corresponding hkl planes at 26.7° (002), 41.6° (100), 43.8° (101), 50.1° (102), 55.0° (004), 71.3° (104), 75.9° (110), and 82.2° (112) as can be seen (Figure 2). This matched to the PDF database (PDF 034-0421) for h-BN showing that the h-BN has not changed its crystal structure with the exfoliation process. Thus, the BNNS retain the crystalline nature of the bulk h-BN, that is similar to previous reports.^21^
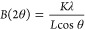
1

The Scherrer equation (eq 1) gives a relation between the peaks full width half maximum (FWHM) and the crystallite size in the material.^22^ Here, L is the crystallite size, K is the Scherrer constant taken as 0.9, λ is the X-ray wavelength, θ is the diffraction angle, and B is the FWHM broadening from the XRD data of a peak. From this equation, it can be seen that as the crystallite size L increases, the line broadening *B* decreases. Each of the peaks in the XRD data was analyzed using the Scherrer equation (Table S1) giving an average of 23.6 nm. There is some discrepancy between the crystallite sizes for individual peaks and the size of the flakes from TEM, but this can be understood as these are 2D flakes, not spherical particles.
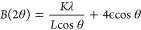
2

The Willimason-Hall method (eq 2) of crystallite size analysis takes into account the contributions to broadening from both size and local strain,^23^ where ε is the strain. The Williamson-Hall method was also applied to the XRD data (Figure S3), and it gave a crystallite size of 8.3 nm, which is different than the Scherrer method. We can see that strain has negligible influence (≈0%) with the broadening coming from size. This discrepancy in size between the two methods can be rationalized again due to the BNNS not being spherical but anisotropic 2D flakes.

**Figure 3:**
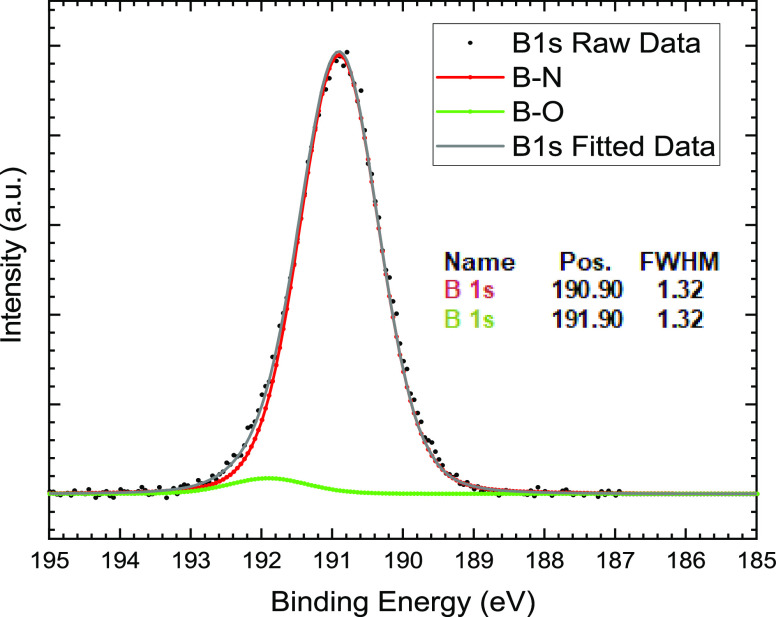
High-resolution XPS of the BNNS B 1s peak showing deconvoluted peaks.

In the XPS survey scan of the BNNS samples (Figure S4), there are peaks corresponding to the B 1s and N 1s core levels at 190.90 and 398.35 eV, respectively, when calibrated to the adventitious C 1s peak at 285.00 eV,^24^ which is similar to our previous result.^25^ C and O are common impurities in XPS samples exposed to the atmosphere. The C impurity, known as adventitious carbon, can be used for calibration of the peak positions.^26^ When constrained to keep the FWHM the same for the deconvoluted peaks, the B 1s peak can be seen to be a combination of two separate peaks in the high-resolution scan of this region (Figure 3). These peaks have previously been assigned to B–N for the major peak at a binding energy of 190.90 eV and B–O for the minor peak at 191.90 eV. Here, the O possibly comes from both terminal oxygens^27^ on the edges of the BN sheet and absorbed O on the BN sheets,^28^ coming from −OH and H_2_O.

FTIR data (see Figure S5 in Supporting Information) showed peaks corresponding to both the powder BN and the exfoliated BNNS at 755 cm^–1^ and a peak at 1300 cm^–1^, similar to previously reported results where these peaks have been attributed to the out-of-plane B–N–B and the in-plane B–N–B vibrations, respectively.^16^

### 2.2 Preparation of BNNS-MNP Nanocomposites

**Scheme 1:**
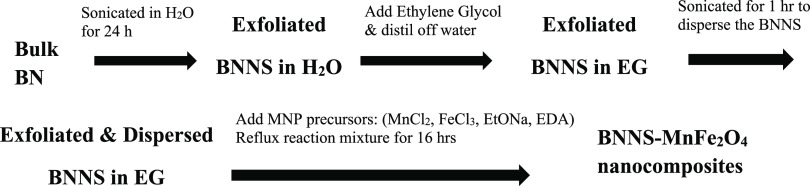
Synthetic Outline for the Creation of the BNNS-MnFe_2_O_4_ Nanocomposites

BNNS-MNP nanocomposites have been synthesized using protocols previously developed by our group.^25^ We prepared BNNS with MnFe_2_O_4_ nanoparticles on the surface of the nanosheets (Scheme 1). In all cases, the procedure involved transfer of the exfoliated BNNS to ethylene glycol for a solvothermal type reaction to form the MNPS in situ on the BNNS. A molar ratio for BN:MnFe_2_O_4_ of 1:0.05 was chosen, giving a ratio of one metal atom per six BN rings, as this ratio was found to give good coverage without separate MNPs observed. The synthesis was carried out multiple times, consistently demonstrating high reproducibility.

In the synthesis, MnCl_2_•4H_2_O and FeCl_3_•6H_2_O serve as precursors for forming the magnetic spinel MnFe_2_O_4_ with the O coming from the hydrated water on the metal chlorides in the basic conditions as OH^–^. These OH^–^ ions form metal hydroxides, which then further react to form the spinel.^29^ EtONa acts as a base, forming ethanol from H^+^ from the water. Ethylenediamine is used as a surfactant as it improves surface coverage. We found that this gave robust attachment of the MNPs to the BNNS even after repeated cycles of sonication and magnetic extraction, the MNPS did not separate from the BNNS. The BNNS-MNP nanocomposites and the corresponding membranes have been characterized by SEM, TEM, FTIR, VSM, XRD, XPS, MIP, and BET techniques.

**Figure 4:**
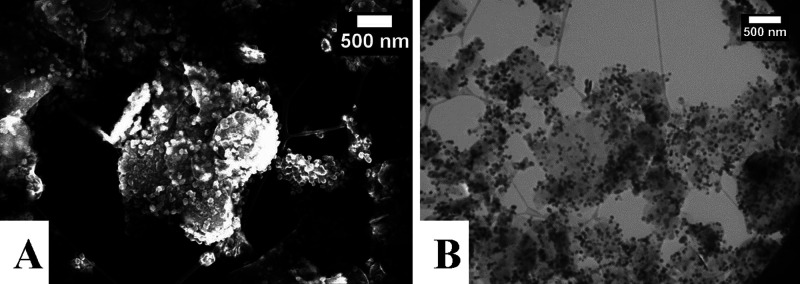
(A) SEM and (B) TEM image of BNNS-MnFe_2_O_4_ nanocomposites.

The TEM and SEM images of the BNNS-MnFe_2_O_4_ nanocomposite are shown (Figure 4). These images confirm the excellent coating of the BNNS with the MnFe_2_O_4_ MNPs, with no MNPs found separated from these sheets. As mentioned previously, the nanosheets varied from around 100–2000 nm in diameter. For the BNNS-MnFe_2_O_4_ nanocomposite, the TEM and SEM images show only the BNNS with the MNPs distributed over the surface. The MNPs range in size from 20 to 80 nm approximately. The particle size distributions of the MNPs were determined by analyzing 100 particles in TEM images through ImageJ software. A size distribution diagram is shown in the Supporting Information (Figure S6).

**Figure 5:**
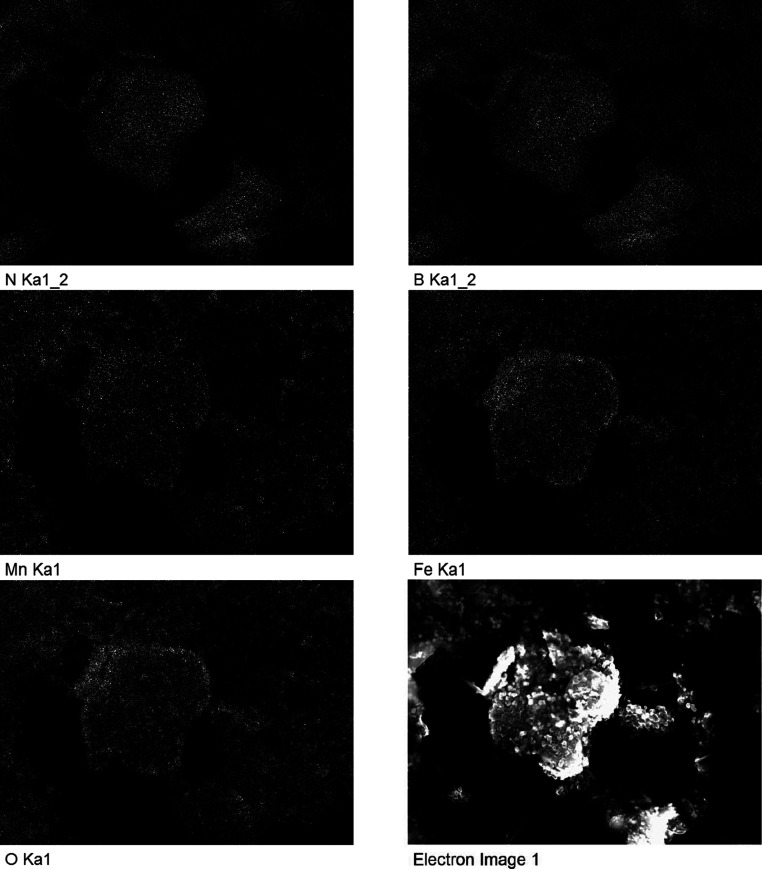
EDX maps of N, B, Mn, Fe, and O showing the detected X-rays from the sample, with the SEM electron image of the sample. From this analysis, we can clearly see that MnFe_2_O_4_ coats the BN sheet.

In the course of SEM analysis, an energy-dispersive X-ray (EDX) detector linked to the SEM was employed to conduct elemental analysis on the BNNS-MnFe_2_O_4_ sample. This was done to elucidate the ratio of elements in the sample and to do elemental mapping of the sample to show where the MnFe_2_O_4_ particles are in relation to the BNNS. For EDX quantification (Figure S7), a substantial sample layer was applied onto carbon stubs, followed by a wide field scan to minimize variations in local coverage. According to the EDX quantification results, the atomic percentages of the Mn:Fe:O:B:N were found to be 1.73:4.52:17.45:38.03:38.27. The ratio of B/N was approximately 1 as expected, while the ratio for Mn:Fe was slightly lower at 0.4 than the theoretical expected value of 0.5. This may indicate some of the Mn(II) ions were washed away in the reaction. The ratio of the metal ions to the O ions (Fe+Mn:O) was 0.36, which was lower than the theoretical expected value of 0.75. This would indicate the presence of adsorbed H_2_O molecules and terminal oxygen atoms on the surface of the nanocomposite. The Mn:Fe:N ratio measured 0.16, surpassing the anticipated value of 0.02. This suggests that during the magnetic extraction process, some of the BN might have been removed. Elemental maps for the BNNS-MnFe_2_O_4_ sample are shown (Figure 5). The images show that the Mn, Fe, and O are evenly distributed on the BNNS to give a good coating of the BN sheet. This supports the SEM and TEM results above (Figure 4), proving that the 2D BNNS are well coated with 0D MNPs.

XRD of these samples was used for identification of the constituent materials because the crystal phase of BN and ferrite spinels has well-defined crystal structures. XRD of the nanocomposites showed peaks for BN with smaller peaks for the various spinels in each of the scans. The boron nitride peaks (e.g., 26.7° (002)) are much larger than the MnFe_2_O_4_ peaks (e.g., 35.0° (311)) because of the ratio of the BN to MNPs in the sample. The PDF-2004 database was used for identifying the phases in the samples.

**Figure 6:**
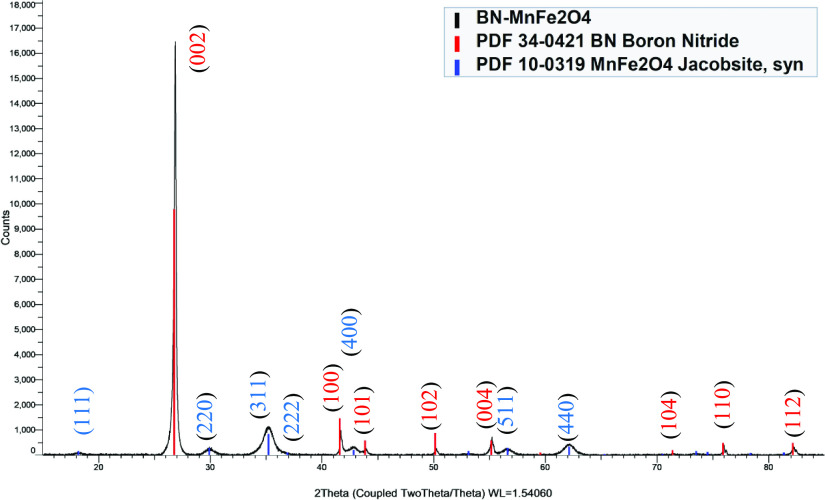
XRD pattern of the BNNS-MnFe_2_O_4_ nanocomposite showing the hkl planes.

For BNNS-MnFe_2_O_4_ (Figure 6), there was only a match for the BN (PDF 034-0421) and MnFe_2_O_4_ (PDF 010-0319) with no other peaks in the scan indicating that this nanocomposite contained only BNNS and MnFe_2_O_4_ in line with the TEM and SEM images. The major peaks at 2θ° and corresponding planes have been assigned for the BN (red) of 26.7° (002), 41.6° (100), 43.8° (101), 50.1° (102), 55.0° (004), 71.3° (104), 75.9° (110), and 82.2° (112) and MnFe_2_O_4_ (blue) of 18.1° (111), 29.7° (220), 35.0° (311), 36.6° (222), 56.2° (511), and 61.7° (440) respectively. This result indicates that the product obtained is phase pure BN and MnFe_2_O_4_ as there are no extra peaks corresponding to other products in the XRD pattern. An analysis of phase quantification (Figure S8) based on the XRD outcome revealed that boron nitride constituted 97.1% of the sample, while MnFe_2_O_4_ accounted for 2.9%. This method of phase quantification assesses the material’s mass percentage within the sample.

Application of the Scherrer equation to the peaks in this two-phase sample gave an average size of 9.02 nm for the MnFe_2_O_4_ MNPs and 33.85 nm for the BNNS (see Table S2 in the Supporting Information). The Williamson-Hall method applied to this two-phase system gave crystallite sizes of 11.6 nm for the MnFe_2_O_4_ MNPs and 21.0 nm for the BNNS with strain in both cases contributing <1% in both cases (Figures S9 and S10). The discrepancy between the BNNS flakes and the crystallite sizes here can be rationalized by the BNNS not having an isotropic morphology, and therefore, the result is an average of the lateral sizes and the thickness of the flakes. For MnFe_2_O_4_, the crystallite size is in good agreement with previous work as the polyol synthesis is known to give particles that are composed of grains^30^ that form the spherical nanoparticle, and the XRD result gives the size of these grains.

VSM analysis of the BNNS-MnFe_2_O_4_ sample gave a magnetization value of 13.1 Am^2^ kg^–1^ at 1 T. There was no residual magnetism when the field was 0 T, with the shape of the magnetization curve indicative of superparamagnetic behavior as there was no coercivity or remanence at 0 T, and the magnetization has not saturated at 1 T, as can be seen (Figure S11 in Supporting Information).

FTIR analysis of the samples (Figure S12 in Supporting Information) gave peaks at 540 cm^–1^ corresponding to the metal-O stretch of the manganese ferrite.^31^ The peaks observed at 755 and 1300 cm^–1^ experienced slight shifts to 780 and 1330 cm^–1^, respectively. This alteration aligns with the anticipated outcome when the ferrite interacts with the BN flakes. This result is also in line with our previous result where for BNNS-Fe_3_O_4_ and BNNS-CoFe_2_O_4_, a similar shift was seen.^25^ These shifts indicate new interactions, which affect the out-of-plane and the in-plane vibration of the BNNS. Previous work has looked at the binding of iron oxides^18^ and Fe ions^32^ to the surface of BN. In the Fe ion study, the group proposed the formation of borazine-metal complex bonds.^32^ The emergence of these fresh interactions is expected to lead to alterations in the IR spectra of BN, corresponding to the observations witnessed in this case. The investigation of iron oxide using DFT calculations demonstrated the optimized binding between iron oxide and the BN sheet, aiming to enhance contaminant adsorption.^18^

**Figure 7:**
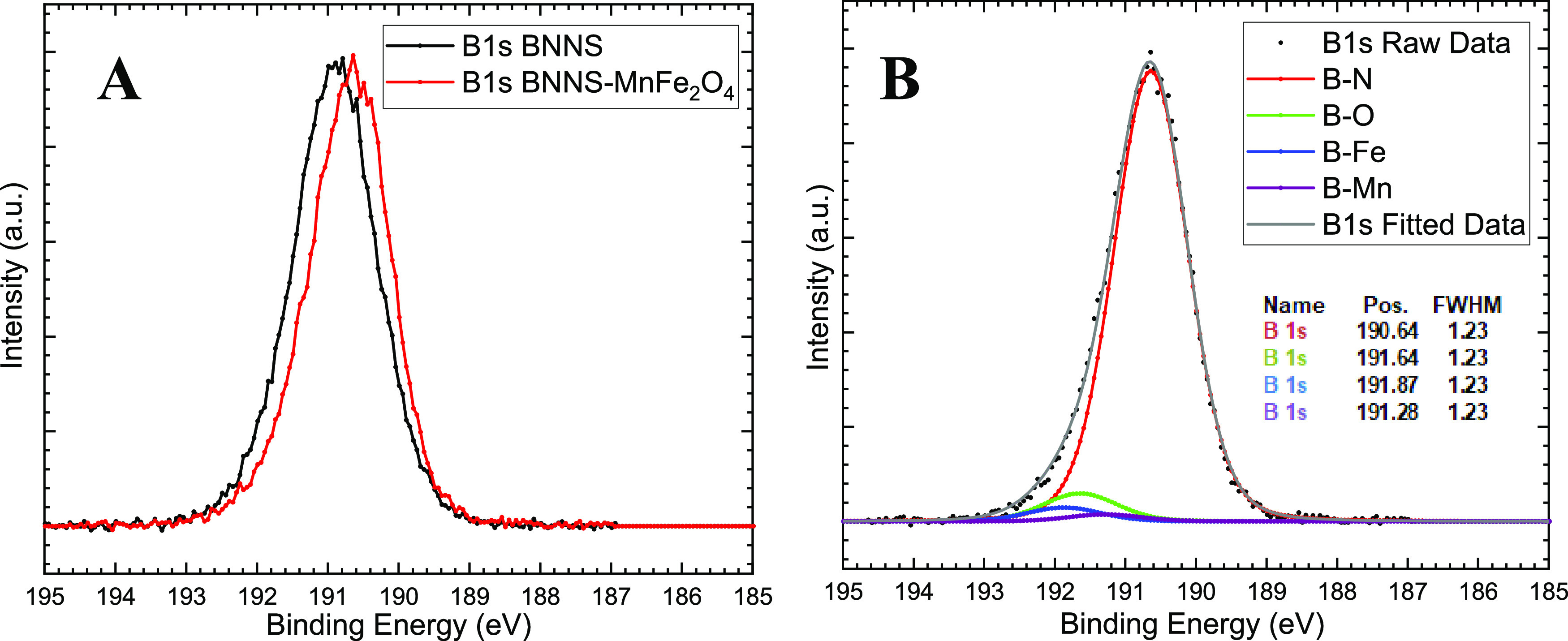
High-resolution XPS spectra (A) B 1s of BNNS and BNNS-MnFe_2_O_4_ composite, (B) B 1s of BNNS-MnFe_2_O_4_ composite showing fitted and deconvoluted peaks. Note the improvement of the fwhm from Figure 3, indicating that there is less chemical disorder of the B sites in this composite.

Within the XPS survey spectrum of the sample (Figure S4), distinct peaks representing B 1s, N 1s, Fe 2p, Mn 2p, and O 1s elements are evident, explaining the elemental composition of the sample. Additionally, a peak corresponding to adventitious C 1s suggests the presence of minor impurities resulting from atmospheric exposure. The adventitious C 1s peak was used for calibration.^26^ In the high-resolution B 1s peak XPS scan (Figure 7A) of the BNNS-MnFe_2_O_4_ sample compared with the BNNS sample, we can see a clear difference in the peak position with a slight asymmetry in the BNNS-MnFe_2_O_4_ compared to the BNNS. Deconvolution of the B 1s peak of the BNNS-MnFe_2_O_4_ sample (Figure 7B) shows it to be a combination of 4 peaks, with the FWHM constrained to be the same for the 4 deconvoluted peaks. We see a shift to a lower binding energy compared with the pure BNNS for the B–N with a peak position of 190.64 eV and B–O with a 191.64 eV. Deconvolution shows that the peak also contain 2 smaller peaks at 191.87 and 191.28 eV that can be attributed to the formation of B–Fe and B–Mn interactions, respectively. The shifting of the B 1s peaks toward higher binding energies upon formation of the B-metal interactions, 191.87 and 191.28 eV respectively, indicates that there is a net movement of electron density from the B sites to the metal centers. The peak position for the B–Mn interaction is at a lower binding energy as compared to the B–Fe contribution, while in our previous paper, the B–Co contribution was at a higher binding energy with respect to the B–Fe interaction.^25^ This can be attributed to the changing electronegativity of Mn (1.55), Fe (1.83), and Co (1.91) with Co showing the largest shift. A decrease in the FWHM, seen in contrast to that of the pure BNNS (1.23 versus 1.32), suggests reduced chemical disorder at the B sites. This shift indicates that as the MNPs adhere to the BNNS through suggested B-metal bond formation, electron density shifts from B to the metal centers. Simultaneously, there is an electron density movement onto the remainder of the sheet likely due to back-donation from the bulk of the ferrite nanoparticles.

To find the optimal ratio of BNNS:MnFe_2_O_4_, different ratios of BNNS to MnFe_2_O_4_ were used in the synthesis, and then, TEM images were taken to view the coverage. The analyzed sample exhibited a BNNS:MnFe_2_O_4_ molar ratio of 1:0.05. Additional nanocomposites were created at ratios of 1:0.1 and 1:0.01 to assess their impact on the coverage and magnetic properties. It was observed that the MNPs’ coverage on the BNNS depended on the BNNS to MnFe_2_O_4_ ratio, with higher molar ratios resulting in greater coverage and lower ratios yielding less coverage. The coverage of the MNPs on the BNNS was found to be dependent on the ratio of BNNS to MnFe_2_O_4_ with the larger molar ratio giving high coverage and the lower ratio giving less coverage. In the high ratio sample, it was found that some separate MNPs formed apart from BN sheets. The TEM images (Figure S13) verified extensive coverage at the 1:0.1 ratio, while the other two ratios exhibited diminishing coverage. Throughout the varying molar ratios, the size of the MNPs on the surface remained consistent, measuring approximately 20–80 nm, as depicted in the images. The BNNSs were all similar sizes from 100 to 2000 nm in all the samples.

The magnetic properties were also dependent on the molar ratio of the nonmagnetic BNNS and the magnetic MnFe_2_O_4_ with the magnetization being 25, 13, and 4 Am^2^ kg^–1^ for the 0.1, 0.05, and 0.01 ratios, respectively. The results of these observations are presented in the Table 1.

**Table 1:** Summary of Product Characteristics for Different Molar Ratios of BNNS:MnFe_2_O_4_

BNNS:MnFe_2_O_4_ molar ratio	coverage of BNNS	magnetization (Am^2^ kg^–1^)
1:0.1	high, some separate MNP	25
1:0.05	medium; no separate MNP	13
1:0.01	low; no separate MNP	4

### 2.3 Membrane Preparation and Surface Area Analysis

**Figure 8:**
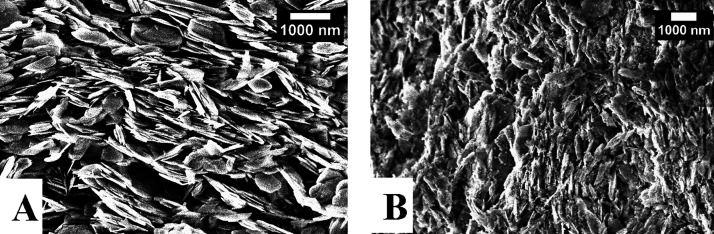
SEM cross sections of (A) BNNS membrane and (B) BNNS-MnFe_2_O_4_ membrane.

BNNS and BNNS-MnFe_2_O_4_ membranes were produced by filtering an aqueous suspension through a 0.45 μm polyvinylidene fluoride (PVDF) filter using a fritted glass filtering apparatus. This method resulted in the formation of nanosheet membranes on the PVDF substrate. To illustrate the layering, SEM images of the edge profile of each membrane were captured. Figure 8 presents these SEM images depicting (A) BNNS and (B) BNNS-MnFe_2_O_4_ in a 1:0.05 ratio. These images showcase the maintained excellent coverage of BNNS coated with MNPs during the membrane formation, demonstrating that the MNPs remained intact without detachment or washing away throughout the process.

BET (Brunauer–Emmett–Teller) analysis provides quantitative data on the specific surface area, with porosity distribution over the range 0–50 nm for solid materials. High pressure MIP (mercury intrusion porosimetry) is a pore size and pore volume analysis technique with a range of 10–10,000 nm. Both methods are suitable for a wide range of particulate and nonparticulate materials. BET and MIP analysis was carried out in close conjunction with adsorption analysis, as adsorption performance is closely related to the specific surface area and pore size distribution of a material.^33^ The surface area was calculated from the linear region of the adsorption branch of the isotherm (*P*/*P*_0_ = 0.1–0.3), while the Barrett–Joyner–Halenda (BJH) method was used to calculate pore size and pore volume from the desorption branch of the isotherm. The BET and MIP analyses were carried out on the BNNS-MnFe_2_O_4_ with a ratio of 1:0.05.

The samples’ surface area depends on the distinct structure of the nanosheets, made up of numerous layers separated by narrow voids. These flakes generally range from 100 to 2000 nm in width, with corresponding minute void spaces between them. Notably, hysteresis appeared in the adsorption–desorption patterns (type IV) for both the uncoated BNNS sample (Figure S14) and the MnFe_2_O_4_-coated BNNS sample (Figure S15 in the Supporting Information).

The BET analysis in the linear region of the isotherm gave a surface area of 32.9 m^2^/g for the BNNS and a surface area of 46.3 m^2^/g for the BNNS-MnFe_2_O_4_. BNNS-MnFe_2_O_4_ has higher surface area relative to the uncoated BNNS which can be attributed to the additional surface area contribution of the MnFe_2_O_4_ nanoparticles. This value was higher than our previously reported surface area values for BNNS-Fe_3_O_4_ (38.8 m^2^/g) and BNNS-CoFe_2_O_4_ (34.6 m^2^/g).^25^

The BJH pore size distributions of the coated and uncoated samples showed similar characteristics; however, the BNNS–MnFe_2_O_4_ had stronger volume adsorption in the region corresponding to pores <5 nm. For uncoated BNNS (Figure S16), the associated pore volume for pores <5 nm was only 3.9% of the total. By contrast, for BNNS–MnFe_2_O_4_ (Figure S17 in the Supporting Information), the pore volume was 11.5% of the total. This is believed to be due to light agglomeration of MnFe_2_O_4_ nanoparticles, resulting in the creation of additional nanometer-scale void spaces, beyond those which are inherently present in the uncoated sample. The results of surface area and porosimetry analysis are summarized in the Table 2.

**Table 2:** Summary of the BET Surface Area and BJH Pore Volume Data

sample	surface area(m^2^/g)	total pore volume (cc/g)	% porosity (<5 nm)
BNNS	32.9	0.18	3.9
BNNS-MnFe_2_O_4_	46.3	0.16	11.5

From MIP analysis for BNNS (Figure S18 in the Supporting Information), a total intruded volume of 0.58 cc/g is observed. The intrusion of mercury corresponds to the filling of void spaces between the BN flakes. A major peak is observed at approximately 100 nm. The intrusion curve then plateaus out before a second sharp intrusion occurs at approximately 20 nm. This peak is due to the void spaces between the smallest BN flakes, ones that are sub-100 nm in width. Clearly, the peak at 100 nm is by far the more prominent one, indicating that the sample is dominated by larger pore spaces, with a subset of smaller pore spaces also present.

For BNNS-MnFe_2_O_4_ (Figure S19 in the Supporting Information), the total intruded volume is 0.70 cc/g. A bimodal pore size distribution is again observed; however, this time, the second peak dominates the distribution, at the expense of the first. This is due to the presence of large numbers of MnFe_2_O_4_ nanoparticles, with void spaces in between. The pre-existing void spaces (ca. 20 nm) between the smallest sub-100 nm flakes detected for BNNSs are also detected for BNNS-MnFe_2_O_4_; they manifest as a broad shoulder on the curve, as they are drowned out by the much larger peak at 50–60 nm. The peak for the void spaces between the largest BN flakes is shifted to the left, indicative of larger void spaces between the flakes, relative to uncoated BNNS. The understanding is that the nanoparticulate coating adheres to the surfaces of the flakes and these attached nanoparticles act to maintain a distance between flakes, which might otherwise come into direct contact. This view is supported by the fact that BNNS-MnFe_2_O_4_ has a larger total intruded volume relative to uncoated BNNS. The coated sample has a higher bulk volume because of the loose packing density of the flakes as well as additional void spaces created by the nanoparticles on the sheets themselves.

### 2.4 Testing the Membranes for Filtration Applications

In our previous work, we had tested the BNNS membrane, the BNNS-Fe_3_O_4_ and BNNS-CoFe_2_O_4_ membrane for removal of methylene blue (MB) from aqueous solution^25^ To test the BNNS-MnFe_2_O_4_ membranes for the retention of MB, membranes with a ratio of 1:0.05 were prepared. A PVDF membrane served as the base for supporting the BNNS-MnFe_2_O_4_ membrane during filtration. When used independently with BNNS-MnFe_2_O_4_ for filtration, the PVDF membrane, positioned on the fritted glass filtering apparatus, exhibited a minimal capability to retain MB, aligning with expectations. Through a calculation involving successive 20 mL portions of the 21.9 μM MB solution until reaching saturation, it was determined that the PVDF membrane, weighing 125 mg, adsorbs 0.1 mg of MB, resulting in an adsorption capacity of 2.5 mg per gram of the membrane. On average, the PVDF membrane measured 0.105 mm in thickness and weighed 125 mg.

The preparation of the BNNS and the BNNS-MnFe_2_O_4_ membrane for testing is described in the experimental section. This gave a membrane with a size of 0.001018 m^2^ (36 mm diameter). For each of the membranes, the BNNS membrane and the BNNS-MnFe_2_O_4_ membrane, a mass of 40 mg of the material was used to create the membrane. The thickness of the membrane, as measured using a micrometer screw gauge was similar to 0.050 mm for the BNNS and 0.053 mm for the BNNS-MnFe_2_O_4_.

Flow rates for the prepared membranes were calculated at one bar pressure using Millipore water (MP H_2_O) filtered through the membrane five times for three different membranes of the BNNS and the BNNS-MNFe_2_O_4_ (see Table S3 in the Supporting Information). The PVDF membrane exhibited a flow rate of 26,700 Lm^–^^2^ h^–1^, surpassing the specified value provided by the membrane manufacturer.^34^ When paired with PVDF, the BNNS membrane achieved a flow rate of 620 ± 53 Lm^–^^2^ h^–1^, while the BNNS-MnFe_2_O_4_ membrane combined with PVDF showed a flow rate of 404 ± 33 Lm^–^^2^ h^–1^. These reductions in flow rates indicate smaller pores in the BNNS-MnFe_2_O_4_ nanocomposite, which has been confirmed with the BET analysis above, where a higher percentage of the porosity was below 5 nm and the nanocomposite had a larger surface area per gram. These membrane characteristics are summarized in Table S4 in the Supporting Information.

The prepared membranes were tested for the retention of MB dye in aqueous solution. The % removal for each aliquot can be calculated using the Beer–Lambert law using ultraviolet–visible (UV–vis) spectroscopy analysis of the filtrate after each 20 mL aliquot. This was done for the BNNS and the BNNS-MnFe_3_O_4_ (Table S5 in Supporting Information). The membranes retained >99% of the MB dye prior to becoming saturated after which MB was found in the filtrate. For the testing, a 40 mg membrane of each material was made on the PVDF support. Subsequently, 20 mL portions of a 21.9 μM MB solution underwent filtration via vacuum filtration using the prepared membranes, resulting in an approximate effective membrane pressure of 1 bar. For the BNNS membrane, 20 mL took 114 s, while for the BNNS-MnFe_2_O_4_ membrane, 20 mL took 175 s. The filtrate initially was clear but began to let the MB through as the membrane became saturated with successive additions of the MB solution.

**Figure 9:**
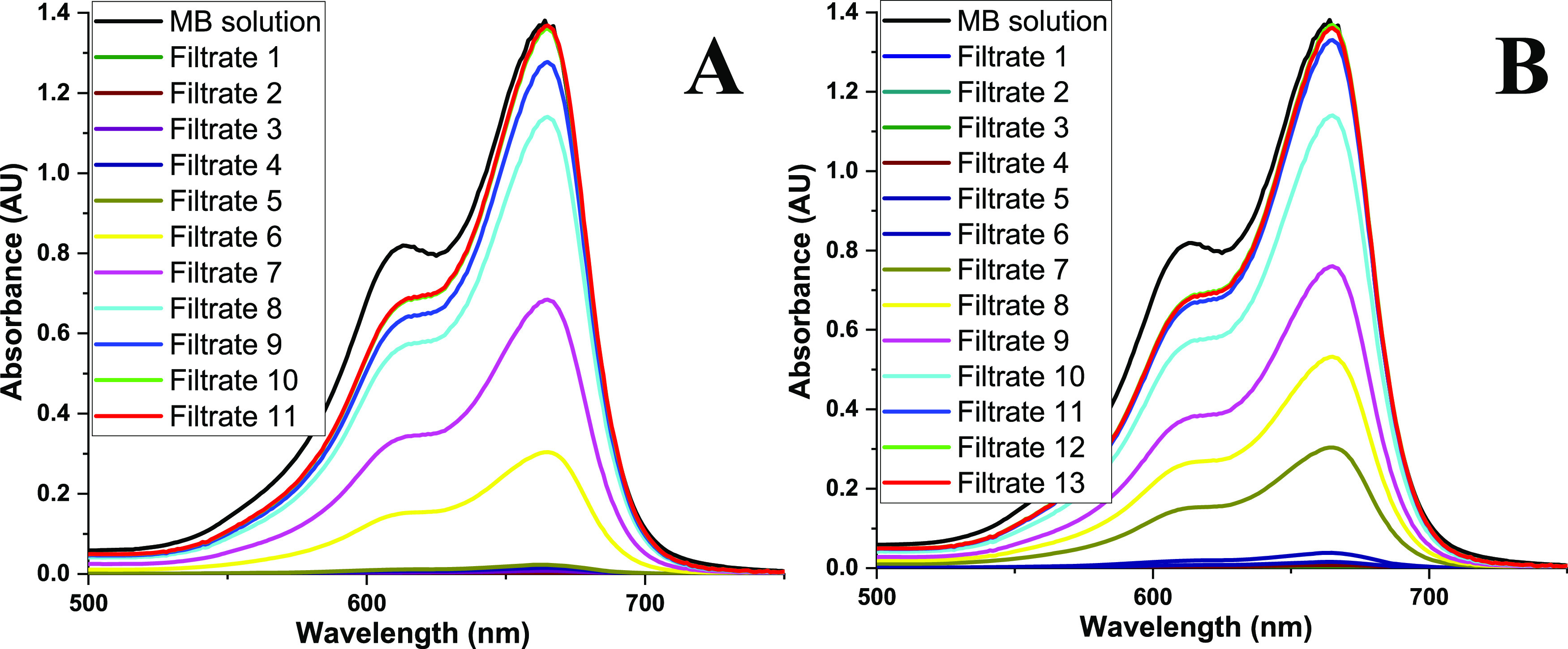
UV–vis analysis of successive 20 mL aliquots of MB solution for (A) BNNS membrane and (B) BNNS-MnFe_2_O_4_. See also Table S5 for numerical values.

The outcomes were assessed using UV–vis spectral analysis specifically for the BNNS membrane, as visualized in Figure 9A. Notably, in the initial few filtrates, over 99% of the dye was successfully removed. However, by the fifth run, the BNNS membrane started allowing MB to pass through as it reached saturation. As filtration continued up to the tenth run, the solution approached the concentration of the original MB solution prior to filtration. At this stage, the membrane had become saturated, hitting its adsorption capacity limit. The same procedure was used to quantify the results for the BNNS-MnFe_2_O_4_ membrane (Figure 9B) with successive 20 mL aliquots of the 21.9 μM MB solution. Here, the membrane began to let dye through on the sixth aliquot and did not reach saturation until the twelfth aliquot.

Twenty mL aliquots of a 21.9 μM MB solution were used with MB having a molar mass of 373.9 g mol^–1^. Each 20 mL will contain 0.164 mg of MB (0.02 L × 0.0219 mM × 373.9 g mol^–1^). From the UV–vis analysis, the total amount of MB absorbed on the membrane can be calculated when the membrane saturates as the graph begins to plateau. The graph shows this for the BNNS and the BNNS-MnFe_2_O_4_ membranes (Figure S20 in the Supporting Information). As can be seen, the BNNS membrane plateaus at 1.07 mg, while the BNNS-MnFe_2_O_4_ membrane plateaus at 1.32 mg. This gives a capture of 1.07 mg for 40 mg of the membrane or 26.75 mg g^–1^ for the BNNS and a capture of 1.32 mg for 40 mg of the membrane or 33.00 mg g^–1^ for the BNNS-MnFe_2_O_4_.

As mentioned earlier, the PVDF membrane captures 2.5 mg g^–1^. Therefore, this value has to be subtracted from the calculated values of the BNNS and BNNS-MnFe_2_O_4_ as these membranes had PVDF membranes as supports. This equates to values of 24.25 mg g^–1^ for BNNS and 30.50 mg g^–1^ for the BNNS-MnFe_2_O_4_. This result clearly demonstrates that the addition of the MnFe_2_O_4_ MNPs to the BNNS gives an increase of 26% in adsorbent capacity. BN adsorption materials with various morphologies have been tested before by various groups for the removal of MB from aqueous solution. These results are summarized and compared to previously reported results for BN based membranes below in the Table 3.

**Table 3:** Adsorption Capacities for Various BN Based Materials from Previous Publications and Our Work

BN material	adsorption (mg g^–1^)	ref
BN spheres	233	(35)
BN nanocarpets	272	(36)
porous BN	313	(37)
BN hollow spheres	117	(38)
BN-MNP aerogel	415	(19)
Activated BN fibers	392	(39)
C doped BNNS	249	(40)
BNNS	24.3	this work
BNNS-Fe_3_O_4_	35.0	(25)
BNNS-CoFe_2_O_4_	21.1	(25)
BNNS-MnFe_2_O_4_	30.5	this work

This work in combination with our previous work^25^ show that MNP can be attached to the BNNS directly without the need for organic linkers. The BNNS and the BNNS-MNP nanocomposites can be used for water filtration, with the addition of the MNP to the BNNS surface increasing the adsorption capacity when MnFe_2_O_4_ MNPs are used. Incorporating MNPS into the BNNS, thus introducing a magnetic aspect, opens up the potential for on-site magnetic inductive heating of the membrane. This feature holds promise for membrane regeneration once it reaches saturation or becomes blocked.

### 2.5 Testing the BNNS-MnFe_2_O_4_ Composites for Regeneration and Recycling

Adsorption of a pollutant onto an adsorbent removes the pollutant from solution but also creates waste in the form of the saturated adsorbent. Recyclability of an adsorbent is essential for sustainable pollutant removal applications.^41^ The BNNS-MnFe_2_O_4_ nanocomposites were tested for recyclability to see if they could be used for filtration applications and then recycled to be used again for the same application without a loss of activity. This testing involved passing the MB solution through a prepared membrane in successive 20 mL portions until the membrane reached saturation. Subsequently, the membrane underwent methanol and water rinses to eliminate excess MB. Following this, the membrane was sonicated in acetone to detach the nanocomposite from the PVDF support. The material was then magnetically separated from acetone and subjected to a 400 °C furnace treatment to eliminate any remaining MB. Afterward, the nanocomposite was sonicated in water to form a new membrane from the same material, which was reused for filtering the MB solution. This recycling process was repeated eight times, and the percentage of MB removed was determined using UV–vis analysis based on the Beer–Lambert law for each cycle. A plot illustrating the adsorption of MB per gram of adsorbent (mg/g) against the recycle number is presented (Figure S21 in Supporting Information). As can be seen, there is not much variability between runs with the second run showing the maximum capture at 31.0 mg/g. There is a general trend of decreasing adsorption but the final two runs show the same capture efficiency at 28.8 mg/g. The same material was used each time and so there was a small loss of material in the process of recycling the membrane. This loss of material was quantified (Table S6), and this was taken into account when calculating the MB saturation adsorption value. TEM and SEM images of the recycled nanocomposites were taken after the 8 recycles to see if the nanocomposite had changed significantly (Figure S22 in Supporting Information). As can be seen in the images, there is no change to the nanocomposite with the MNPs still attached to the surface of the BNNS. TGA of the nanocomposite was performed prior to MB adsorption and with MB adsorbed (see Figures S23 and S24 in Supporting Information). As can be seen from the TGA curves, there is a large change in mass up to 200 °C which can be attributed to removal of adsorbed water. After this, there is the burning off of any organic pollutants. For the MB adsorbed nanocomposite, there is a larger percent change of 94.8% compared to 97.5%. This agrees with our previous analysis where the adsorbed MB makes up 3% of the mass (30.5 mg/g). The attachment of the MnFe_2_O_4_ magnetic nanoparticles to the surface of the BNNS causes an improvement in the adsorption efficiency of 26% compared to the BNNS alone. The excellent performance regarding the adsorption of MB and recyclability shows that this material has potential application in a water purification system. The addition of the magnetic functionality to the BNNS gives the possibility of magnetic inductive heating regeneration of the membrane. Further studies will be performed to test this as a potential property of this nanocomposite.

## 3 Experimental Section

### 3.1 Materials

Iron(lll) chloride hexahydrate, reagent grade ≥98%; manganese(ll) chloride tetrahydrate, reagent grade ≥98%; ethylene glycol, anhydrous 98%; methylene blue, reagent grade; ethanol, HPLC grade; and boron nitride, reagent grade were supplied by Sigma-Aldrich. The Durapore 0.45 μm PVDF membrane was supplied by Merck. Ethylenediamine, >99% was supplied by Merck-Schuchardt. Sodium hydroxide, reagent grade was supplied by Fisher Scientific. MP H_2_O (Millipore water) was acquired through a Synergy 185 Millipore filtration system employing a 0.22 μM filter designed for purifying distilled water. Technical-grade solvents, >99% pure (Ethanol, Methanol, and Acetone), provided by Lennox, were utilized for washing and storage purposes.

### 3.2 Instrumentation

The **TEM** instrument employed for this study was the JEOL 2100 instrument, operating at 200 kV. Meanwhile, the **SEM** utilized was the Zeiss Ultra plus SEM, capable of an accelerating potential ranging from 30 to 1 keV. To capture the images, the SEM was operated within a range of 15–2 keV. **EDX** analysis was conducted on the SEM, employing an Oxford Instruments 80 mm^2^ XMAX EDX detector while operating the SEM at 15 keV. The sample for EDX analysis was positioned on Lacey carbon TEM grids, mounted on a holder within the SEM, focusing specifically on individual MNP coated BN flakes. **UV–vis** spectra were gathered using the Agilent Cary 60 spectrophotometer, covering a range from 1100 to 190 nm and a quartz cuvette with a path length of 1 cm. For **pXRD** analysis, the Bruker D2 Phaser second generation powder sample X-ray machine was utilized, equipped with monochromatic high-intensity Cu Kα radiation (λ = 0.15406 nm). The XRD data collected were background subtracted, spanning 2θ angles from 15° to 85°. Magnetization measurements of the dry products were obtained using an in-house assembled **VSM** at room temperature, applying a field up to 1.1 T. Calibration of the VSM was carried out by employing a pure nickel sample with a known mass. Nickel, being a ferromagnetic material, possesses a recognized magnetic moment of 55.4 Am^2^kg^–1^ in an external field of 1 T at room temperature. **FTIR** spectra were acquired using a PerkinElmer spectrum 100, fitted with a diamond window covering a range from 4200 to 250 cm^–1^. For the **BET** surface area analysis, a Nova 2400e surface area analyzer (Quantachrome, UK) employing nitrogen gas as the adsorbate was utilized. Prior to analysis, the sample underwent a degassing process at 200 °C under vacuum for 1 h. **XPS** measurements were conducted utilizing an Omicron EA 125 Energy Analyzer, employing a monochromated Al K-alpha source at 1486.7 eV. High-resolution core level XPS scans were performed with a pass energy of 20 eV, using high magnification mode and entrance and exit slits of 6 and 3 mm, respectively, resulting in an overall source and instrument resolution of 0.6 eV. **MIP** (Mercury Intruded porosimetry) analysis was conducted using an Autoscan-33 porosimeter (Quantachrome, UK).

### 3.3 Preparation of BNNSs

BNNSs were synthesized from bulk BN using a method described in our previous publication.^5^ A mixture of 300 mg of bulk BN powder and 100 mL of ultrapure water was placed in a 150 mL round-bottom flask. The solution underwent 24 h of sonication using a Wise Clean WUC-A03H operating at 40 kHz with an output of 124 W. Subsequently, this solution was directly employed for transfer into ethylene glycol.

### 3.4 Preparation of EtONa

An 85 mL quantity of anhydrous ethanol (1.47 mol) underwent degassing and was placed under argon in a 250 mL round-bottom flask. Subsequently, 5.87 g of sodium hydroxide (0.15 mol) was introduced, and the mixture was stirred magnetically under argon until fully dissolved. Following this, 26.45 g of 300-mesh molecular sieves was added, and the RBF was sealed under an argon atmosphere. The solution was left undisturbed for 48 h. The liquid phase was separated from the molecular sieves under an argon atmosphere and then distilled to eliminate excess ethanol, resulting in a dry white powder of sodium ethoxide (EtONa). Ethylene glycol (100 mL), pre-degassed, was added to form a solution with a concentration of 0.1 g/mL.

### 3.5 Transfer of BNNSs from Water to Ethylene Glycol

300 mg of BNNS (Boron Nitride Nanosheets) dissolved in 100 mL of water was combined with 120 mL of ethylene glycol in a 500 mL round-bottom flask and stirred using a magnetic stirrer. The water was distilled and gathered; the distillation ceased when 100 mL of water was added. Afterward, the solution was cooled to room temperature and subjected to 1 h of sonication to evenly disperse the nanosheets throughout the ethylene glycol.

### 3.6 Preparation of BNNS-MnFe_2_O_4_

100 mg of BNNSs, equivalent to 4 mmol of BN, was dissolved in 40 mL of ethylene glycol within a 100 mL round-bottom flask. Subsequently, a mixture of FeCl_3_·6H_2_O (0.109 g, 0.40 mmol), MnCl_2_·4H_2_O (0.039 g, 0.2 mmol), and ethylenediamine (0.40 mL, 6.0 mmol) was added, and the solution was sonicated for 30 min in an open-air environment. To this, a solution of EtONa (0.55 g, 8 mmol) in 5.5 mL of ethylene glycol was introduced. The resultant solution was mechanically stirred for 30 min at room temperature to ensure thorough mixing before undergoing reflux for 16 h in an open-air setup. After cooling to room temperature, the particles were separated using a magnetic process, washed twice with water (100 mL each) and ethanol (100 mL each), and finally stored in 100 mL of ethanol.

### 3.7 Preparation of Boron Nitride Nanosheet–Magnetic Nanoparticle Nanocomposite Membranes

A solution of BNNS-MnFe_2_O_4_ (40 mg) in ultrapure water (100 mL) underwent a 2 h sonication process to ensure complete dispersion of the material. Subsequently, the solution was filtered using a PVDF 0.45 μM filter on a fritted glass setup to form the membrane. The freshly formed membrane, with the PVDF filter in place on the fritted glass apparatus, was immediately utilized for filtration without allowing it to dry out. However, for measurements pertaining to mass and thickness, a newly prepared membrane was allowed to dry before conducting these assessments.

### 3.8 Testing of the Membrane for Extraction of Dye

A new membrane of 40 mg, composed of the specified material (BNNS or BNNS-MnFe_2_O_4_), was created. A solution containing MB (20 mL, 21.9 μM) was passed through the membrane, and the resulting filtrate was collected for subsequent UV–vis measurements. This process was iterated with equal volumes of solution until the membrane reached saturation.

### 3.9 Testing the BNNS-MnFe_2_O_4_ for Regeneration and Recycling

An utilized BNNS-MnFe_2_O_4_ membrane underwent rinsing with methanol and water until the filtrate became clear. Subsequently, the membrane was sonicated in a small quantity of acetone to detach the PVDF support. The material was separated using a magnetic process from acetone and subsequently subjected to a 400 °C furnace treatment. The resulting material was sonicated to reintroduce it into a solution of ultrapure water (100 mL). This solution, containing the material, was then utilized to generate a new membrane and repeat the experiment.

## 4 Conclusions

Thus, we have prepared new BNNS functionalization with MnFe_2_O_4_ magnetic spinel ferrite nanoparticles. The TEM and SEM images for this sample showed good coverage at a BNNS:MnFe_2_O_4_ molar ratio of 1:0.05. FTIR and XPS analyses have indicated the formation of B-metal bonds between the MnFe_2_O_4_ and the BNNS. The magnetic properties of the BNNS-MnFe_2_O_4_ nanocomposite were sufficiently good with the sample capable of being extracted from solution with a permanent neodymium magnet. Surface area analysis and high pressure mercury intrusion porosimetry were performed on BNNS and BNNS-MnFe_2_O_4_ nanocomposites. This showed that the surface area increased upon the addition of the MNPs to the BNNS surface. The BNNS and BNNS-MnFe_2_O_4_ membranes were evaluated for their efficiency for removing MB dye from water. We have conducted filtration experiments using MB solution and quantified the membrane’s retention ability through UV–vis spectroscopy analysis. Both membranes retained over 99% of MB until saturation. The BNNS-MnFe_2_O_4_ membrane captured 26% more methylene blue than the BNNS membrane, with adsorption calculations quantifying this result. Comparisons with prior research demonstrated the effectiveness of the BNNS-MnFe_2_O_4_ membrane in MB removal. The nanocomposites were tested for their ability to be recycled without a loss of efficiency. The nanocomposite was recycled eight times. The BNNS-MnFe_2_O_4_ nanocomposite demonstrated consistent performance for MB removal with a slight decrease over runs. The nanocomposite’s attachment of MnFe_2_O_4_ nanoparticles improved adsorption efficiency compared to the BNNS alone. The combination of high MB adsorption and recyclability suggests great potential for water purification systems, and the addition of magnetic functionality for inductive heating regeneration holds promise for advanced future exploration.

## Supporting Information Available

TEM and SEM images of blank Lacey carbon grids; particle size distribution charts; Scherrer analysis tables; Williamson-Hall plots; FTIR spectra of BN powder and exfoliated BNNS; XPS survey spectra of the BNNS, MnFe_2_O_4_, and BNNS-MnFe_2_O_4_; XRD phase quantification of BNNS-MnFe_2_O_4_; EDX of the BNNS-MnFe_2_O_4_; VSM of the BNNS-MnFe_2_O_4_; FTIR of the BNNS-MnFe_2_O_4_, MnFe_2_O_4,_ and BNNS; TEM images of the BNNS-MnFe_2_O_4_ at different ratios; nitrogen adsorption–desorption isotherms; BJH pore size distributions; MIP results; saturation graphs for membranes; Flow rate tables; recycling results for BNN-MnFe_2_O_4_; and TEM and SEM image of BNNS-MnFe_2_O_4_ after 8 recycles (PDF)
