# Supplementary material for: Boron Nitride Nanosheet–Magnetic Nanoparticle Composites for Water Remediation Applications
Source: ACS Omega. 2024 Jan 15;9(4):4347–58. doi: 10.1021/acsomega.3c06593 (PMC10832022; doi:10.1021/acsomega.3c06593)
Supplement: Supplementary file 1 — ao3c06593_si_001.pdf [file ao3c06593_si_001.pdf]

Supporting Information

Boron Nitride Nanosheet – Magnetic Nanoparticle Composites for Water Remediation Applications

Garret Dee[α], Olivia O'Donoghue[α], Eoin Devitt[α], Tiphaine Giroud[β], Aran Rafferty[α], Lee Gannon[γ], Cormac McGuinness[γ] and Yurii K. Gun'ko[α]*

[α] School of Chemistry, University of Dublin, Trinity College, Dublin 2, Ireland

[β] SIGMA Clermont, Campus De Clermont-Ferrand, 63178 AUBIERE CEDEX, France

[γ] School of Physics University of Dublin, Trinity College, Dublin 2, Ireland

Corresponding Author

*Yurii K. Gun'ko - E-Mail: igounko@tcd.ie;

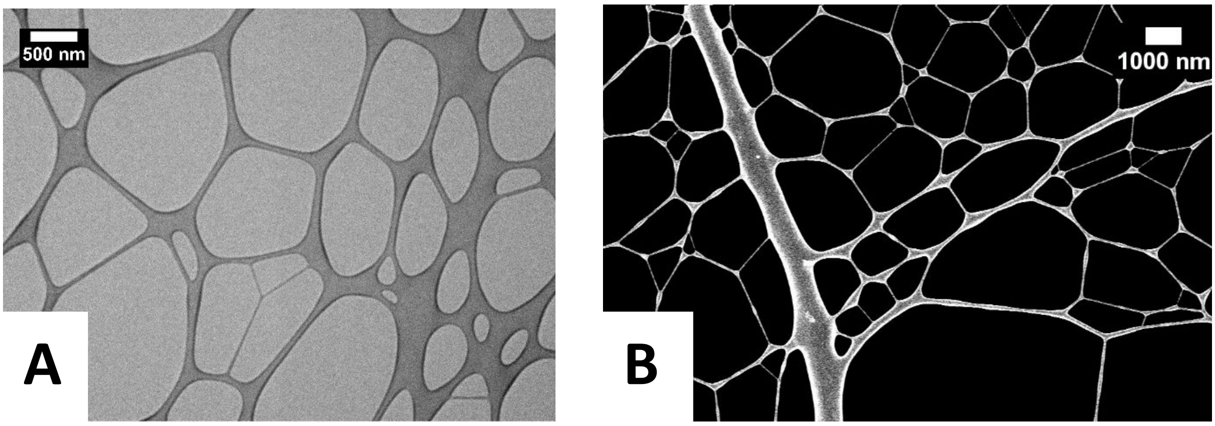

Figure S1: (A) TEM image and (B) SEM image of a blank Lacey carbon grid showing the carbon filaments.

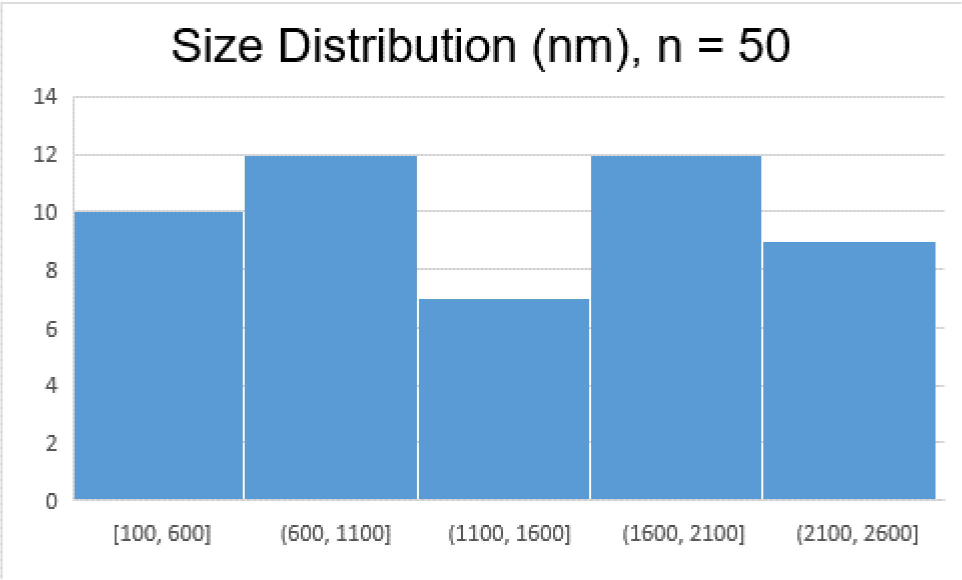

Figure S2: Size distribution for the BNNS flakes for length and width

Table S1: Scherrer analysis result for the individual peaks from XRD of the BNNS

peak position 2θ (°)	FWHM β (°)	D (nm)
26.6918	0.3181	25.67
41.6157	0.329	25.83
43.765	0.648	13.21
50.071	0.727	12.06
55.0483	0.351	25.52
59.47	0.95	9.63
71.336	0.318	30.75
75.886	0.265	38.01
82.122	0.329	32.02
Average		23.6

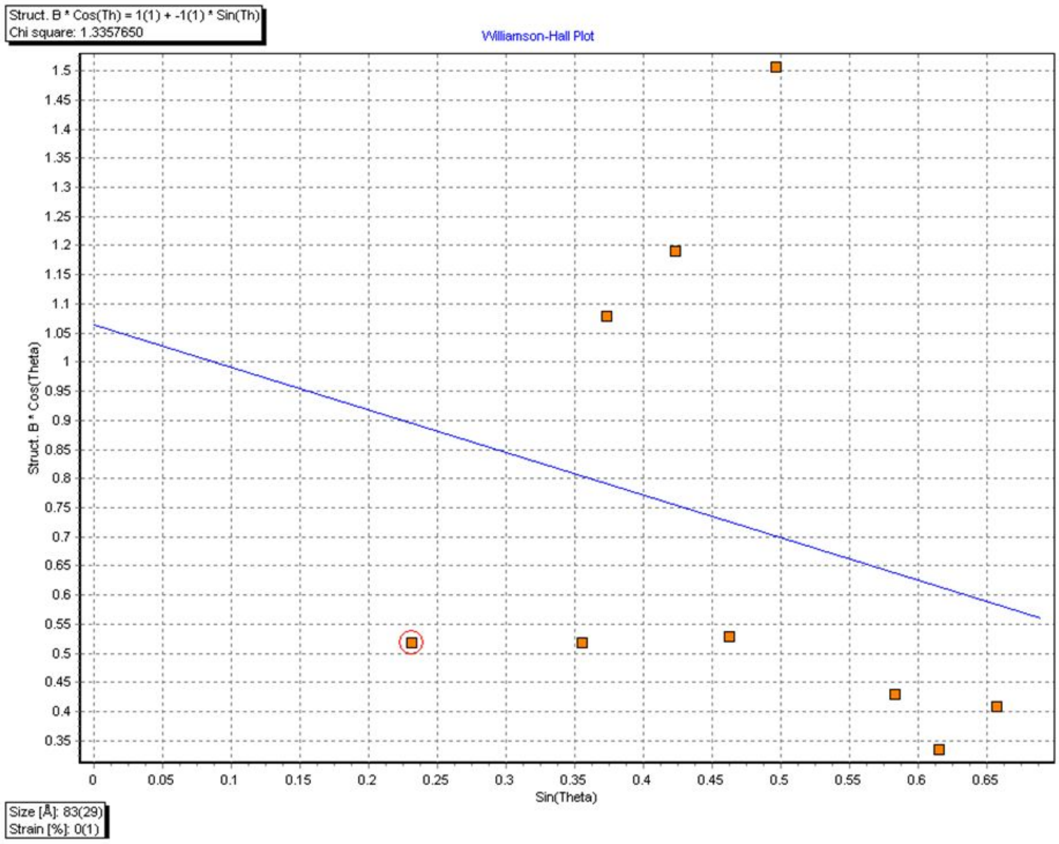

Figure S3: Williams-Hall plot from XRD of the BNNS.

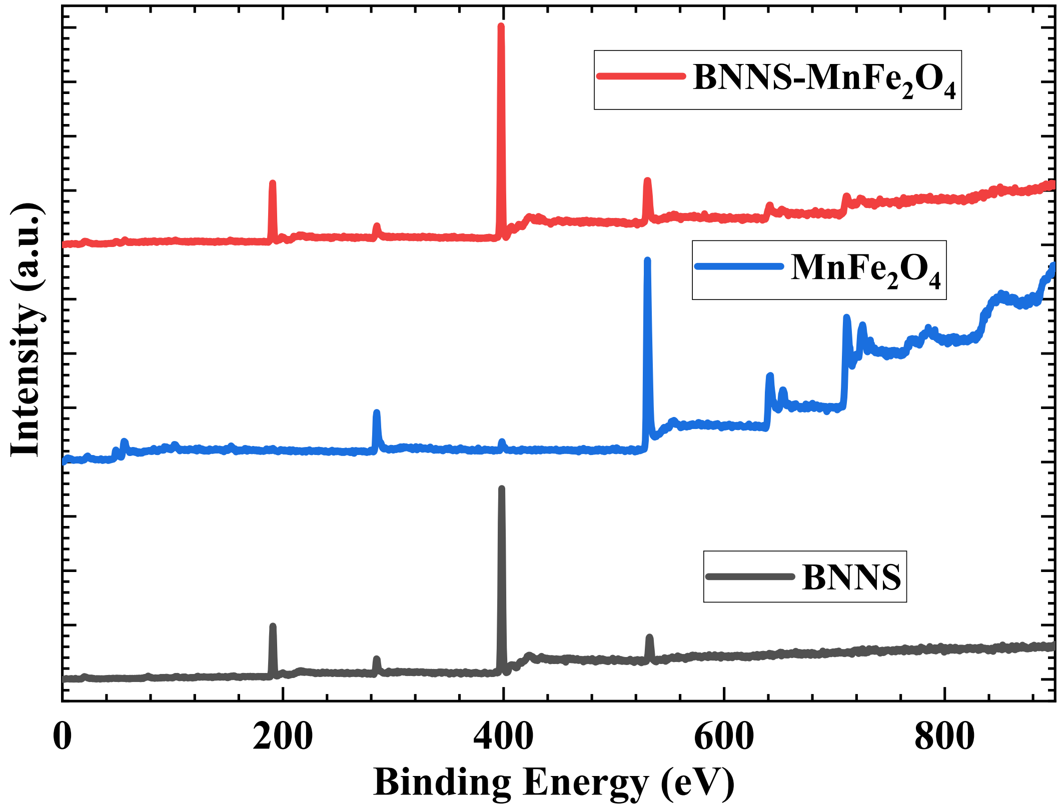

Figure S4: XPS survey spectra of the BNNS, $MnFe_2O_4$ and BNNS-$MnFe_2O_4$.

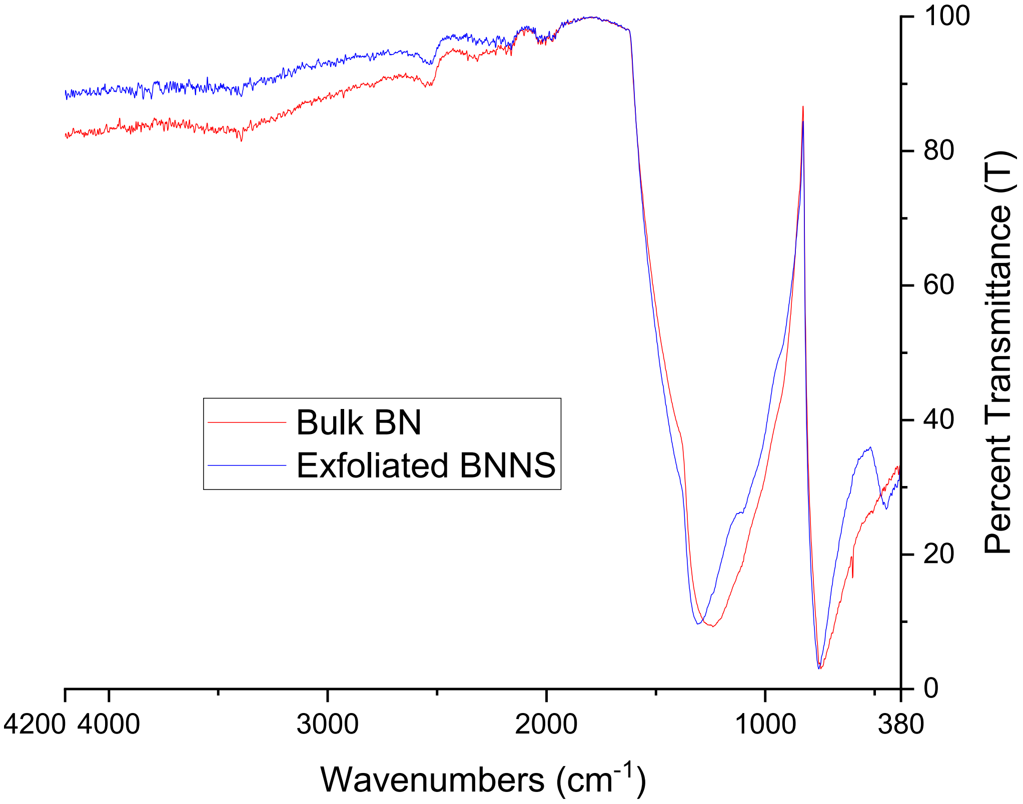

Figure S5: FTIR of BN powder and exfoliated BNNS.

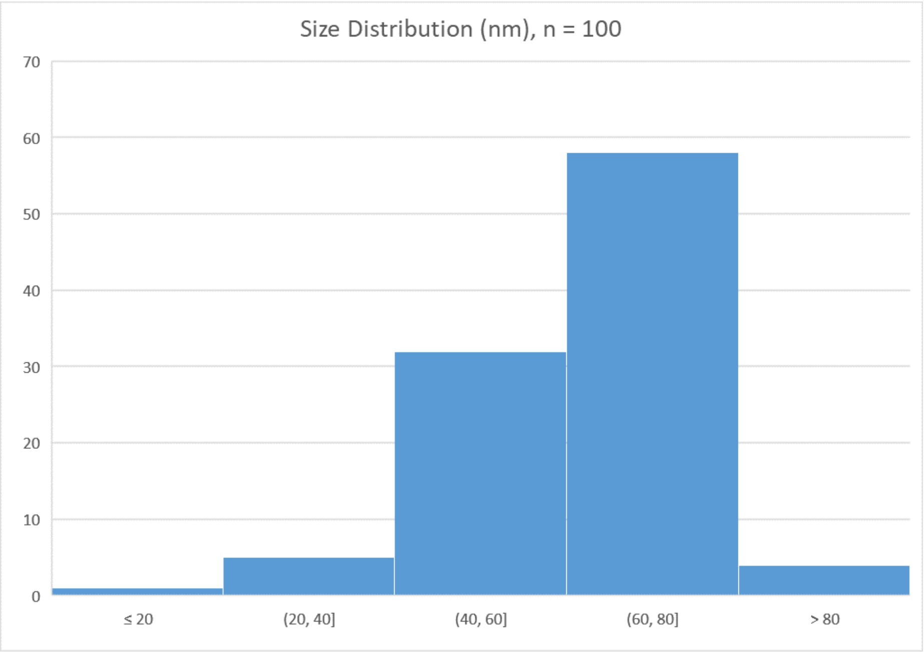

Figure S6: Size distribution for the $MnFe_2O_4$ nanoparticles on the surface of the BNNS.

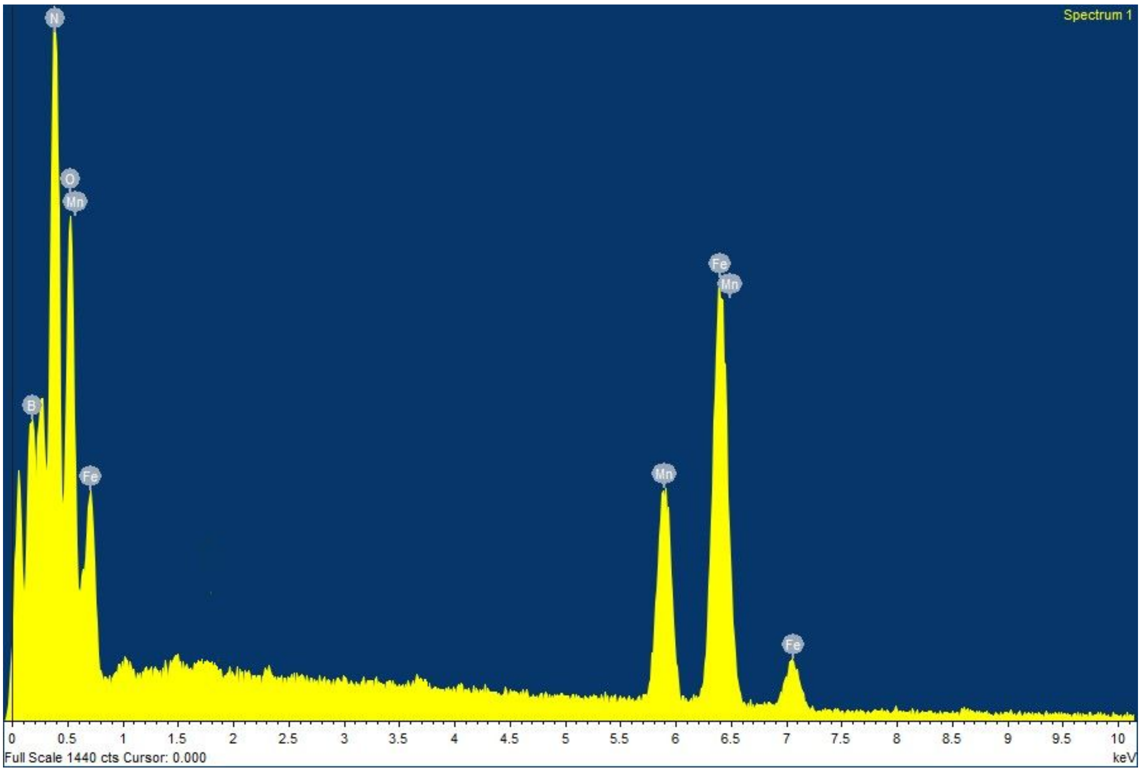

Figure S7: EDX of the BNNS-$MnFe_2O_4$ nanocomposite.

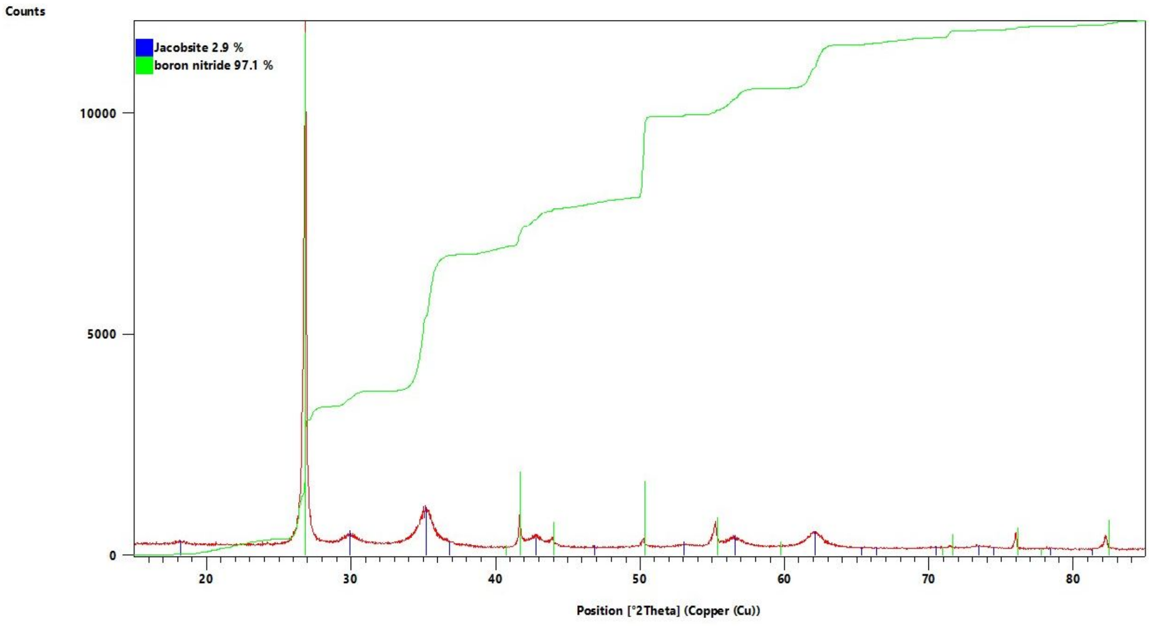

Figure S8: XRD phase quantification result for the BNNS-$MnFe_2O_4$.

Table S2: Scherrer analysis of the Peaks in the BNNS-$MnFe_2O_4$ composite

BNNS peaks			$MnFe_2O_4$ peaks		
peak position 2θ (°)	FWHM β (°)	D (nm)	peak position 2θ (°)	FWHM β (°)	D (nm)
18.12	0.51	15.774	26.8225	0.209	39.077
29.849	0.85	9.673	41.673	0.188	45.214
35.163	0.87	9.579	43.87	0.47	18.222
42.71	0.88	9.693	50.198	0.37	23.710
53	1.33	6.674	55.182	0.268	33.447
56.62	1.68	5.371	71.44	0.32	30.578
62.077	1.06	8.747	75.961	0.2	50.394
73.63	1.5	6.615	82.187	0.35	30.118
average		9.02	average		33.85

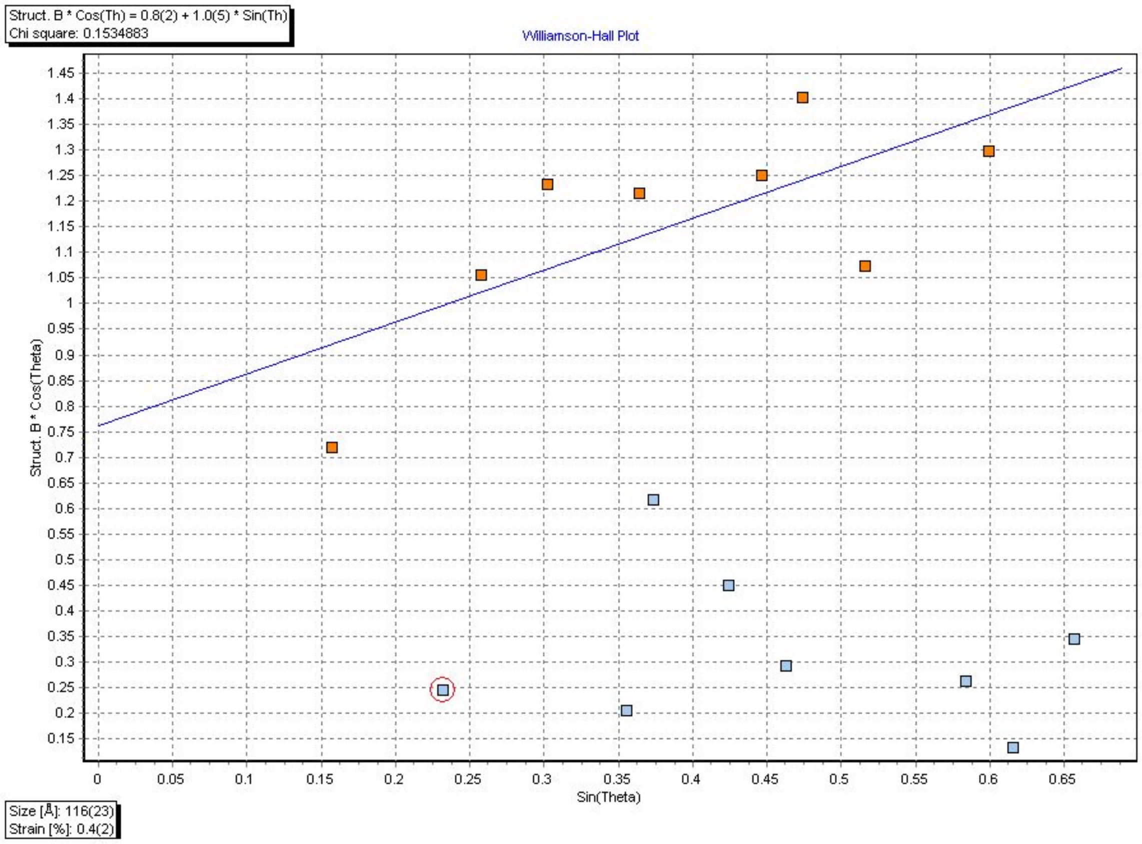

Figure S9: Williamson-Hall plot for the $MnFe_2O_4$ MNPs peaks.

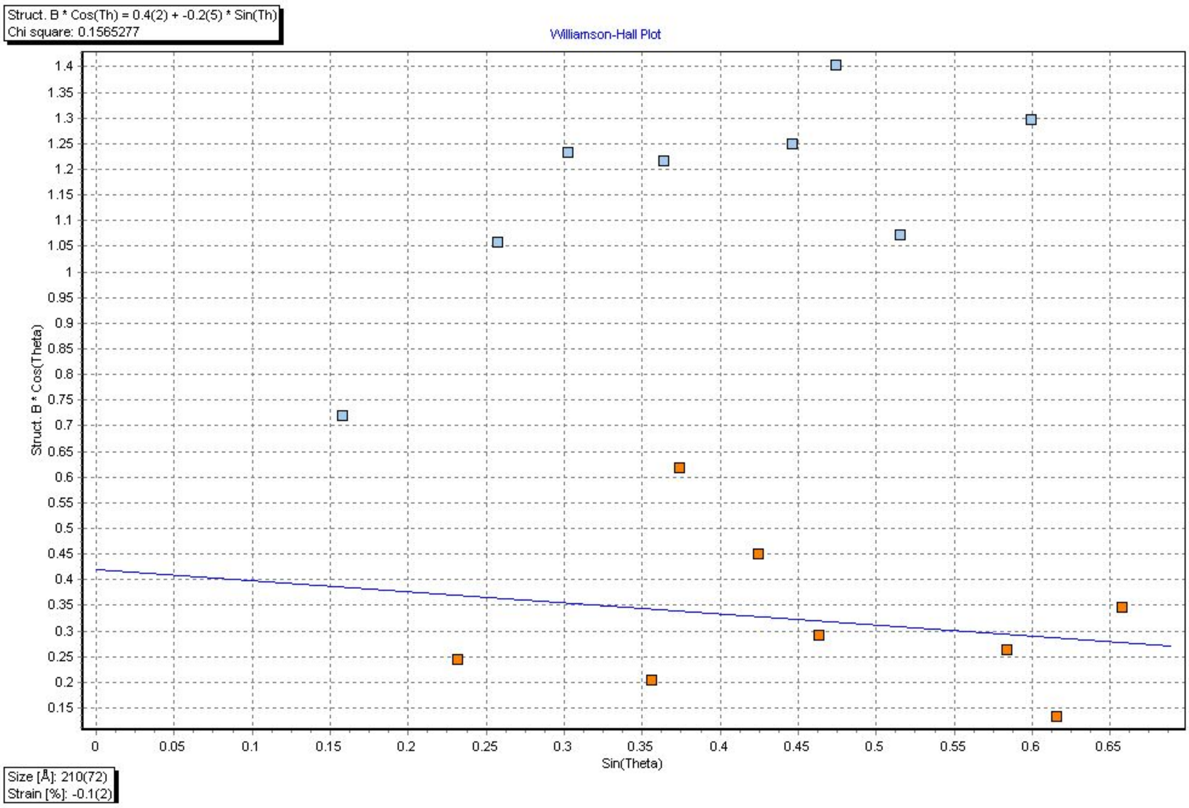

Figure S10: Williamson-Hall plot for the BNNS peaks.

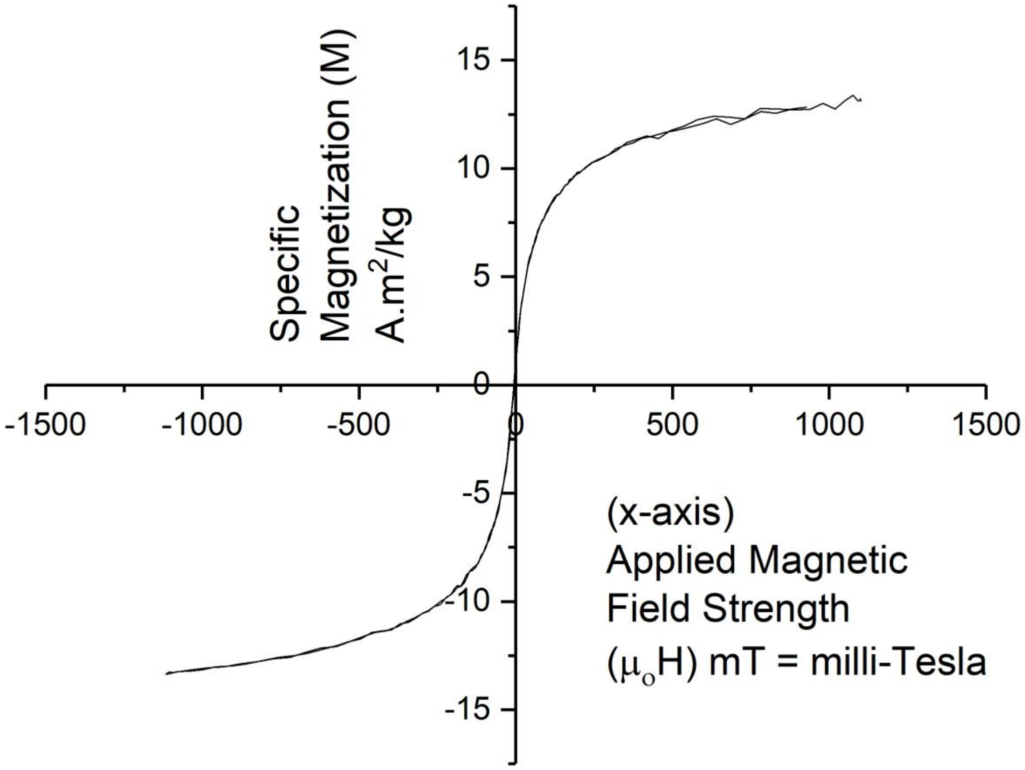

Figure S11: VSM hysteresis loop of theBNNS-$MnFe_2O_4$ nanocomposite.

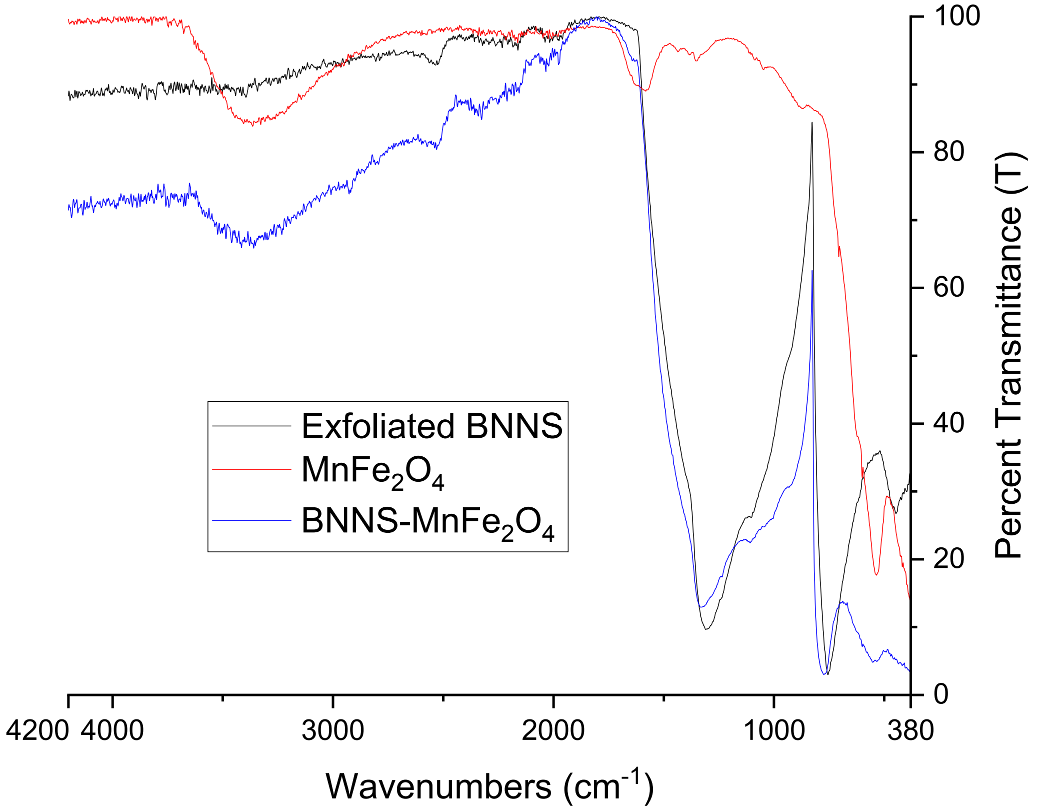

Figure S12: FTIR spectra of BNNS-$MnFe_2O_4$ with BNNS and $MnFe_2O_4$ for comparison.

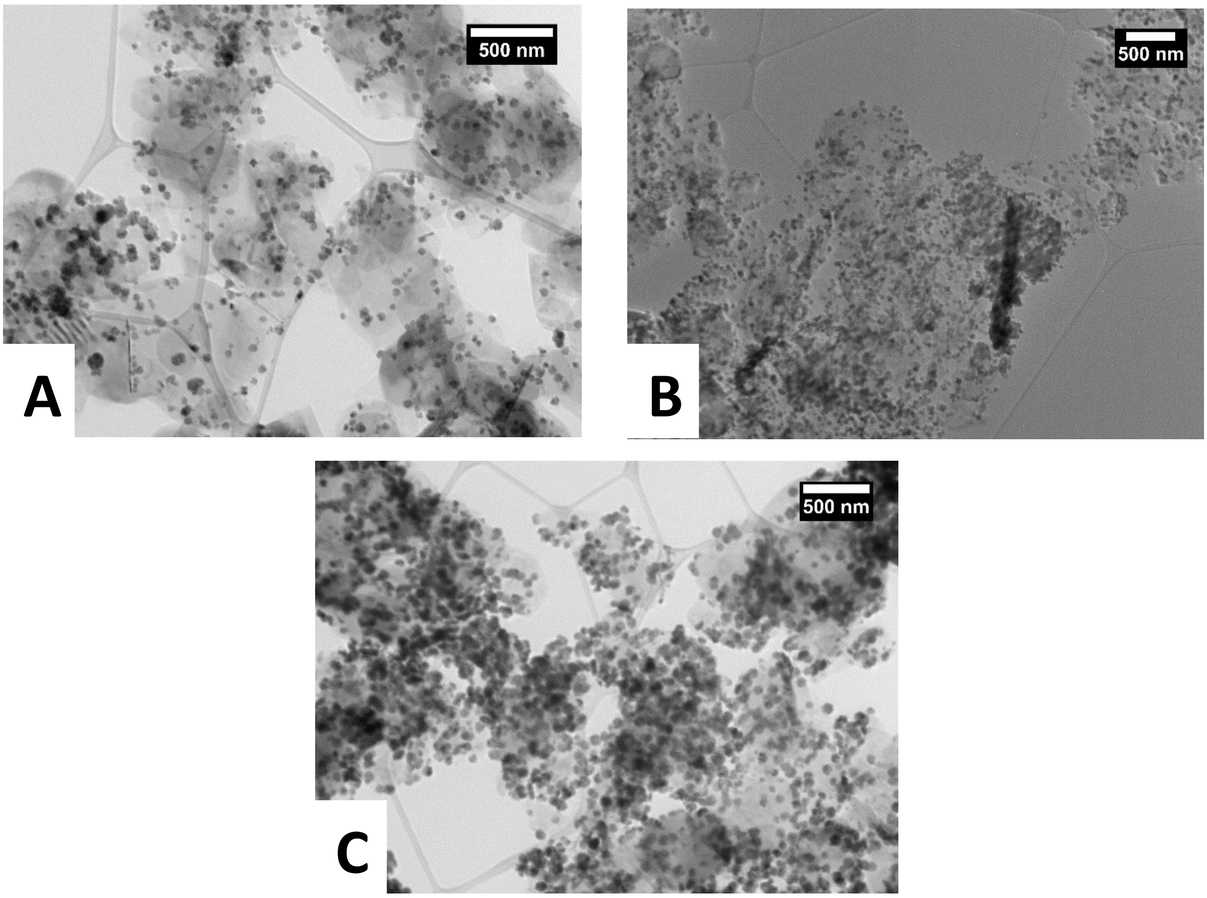

Figure S13: TEM images of the BNNS-$MnFe_2O_4$ with molar ratios of (A) 0.01 (B) 0.05 (C) 0.1

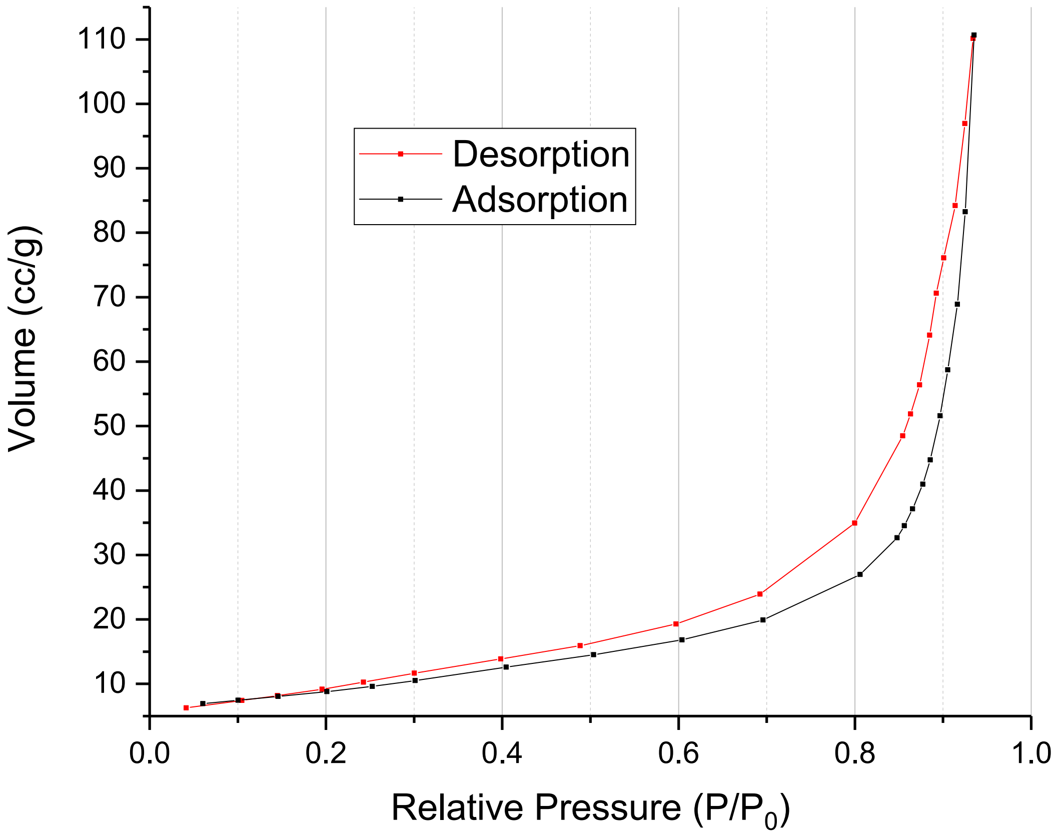

Figure S14: Nitrogen adsorption-desorption isotherm for the BNNS sample.

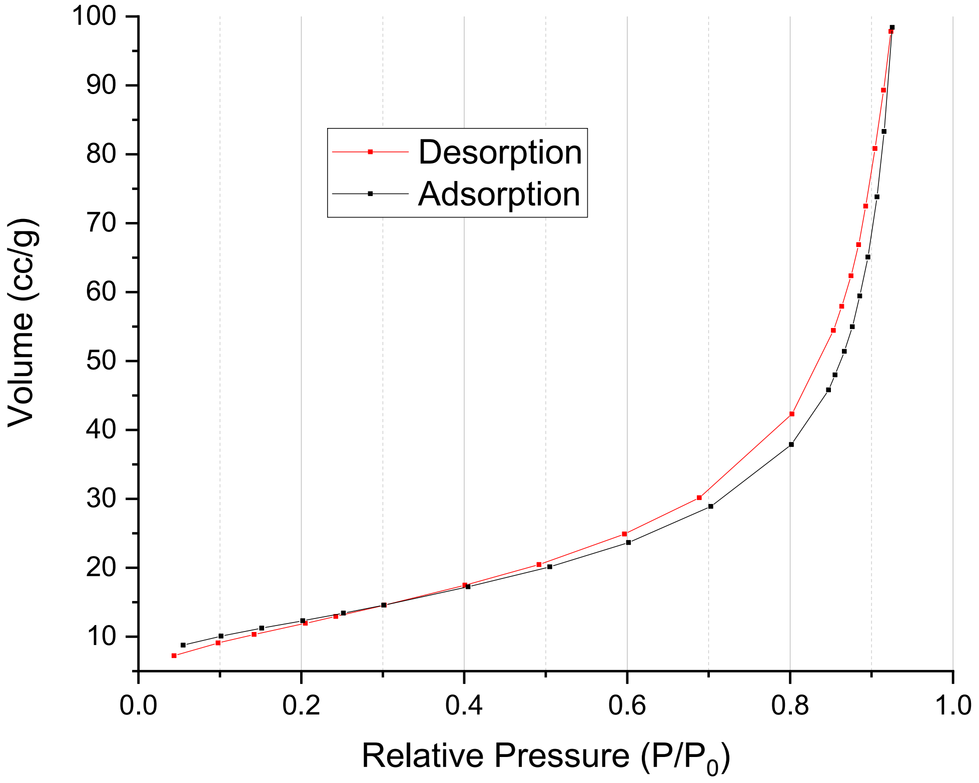

Figure S15: Nitrogen adsorption-desorption isotherm for the BNNS-$MnFe_2O_4$ nanocomposite.

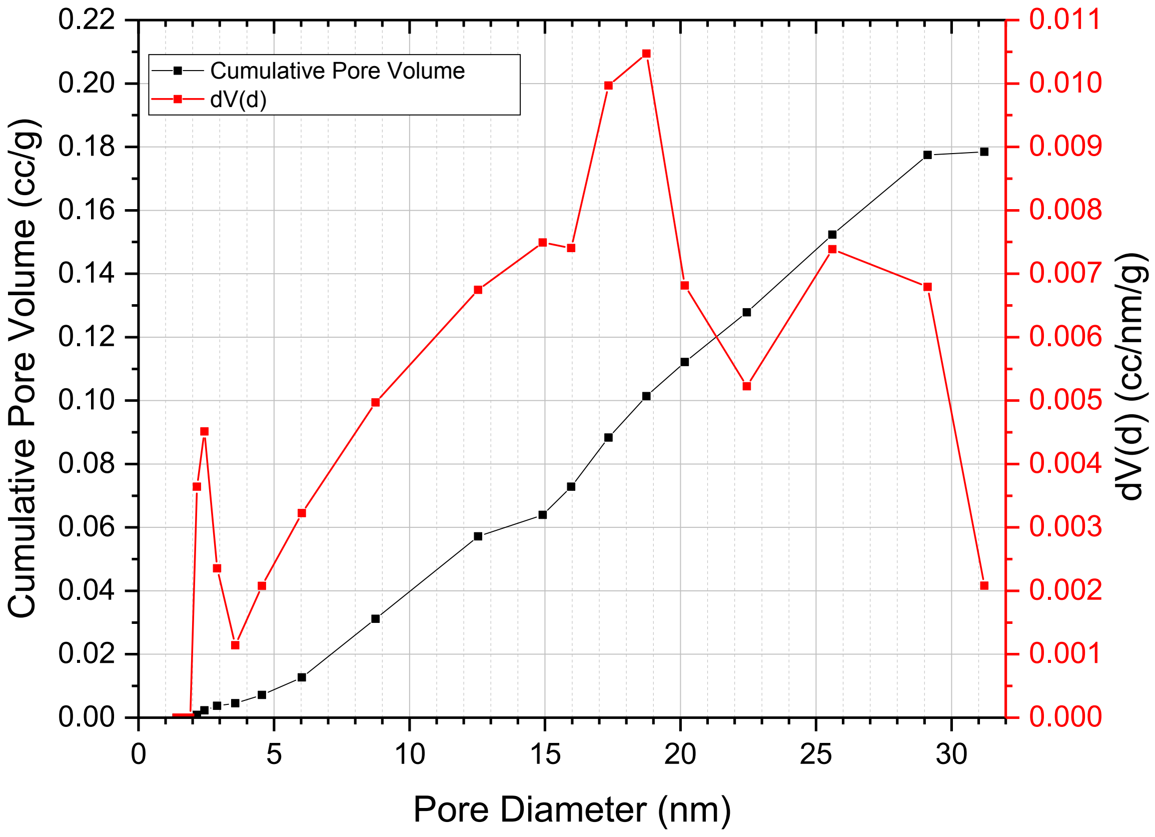

Figure S16: BJH pore size distribution of the BNNS sample.

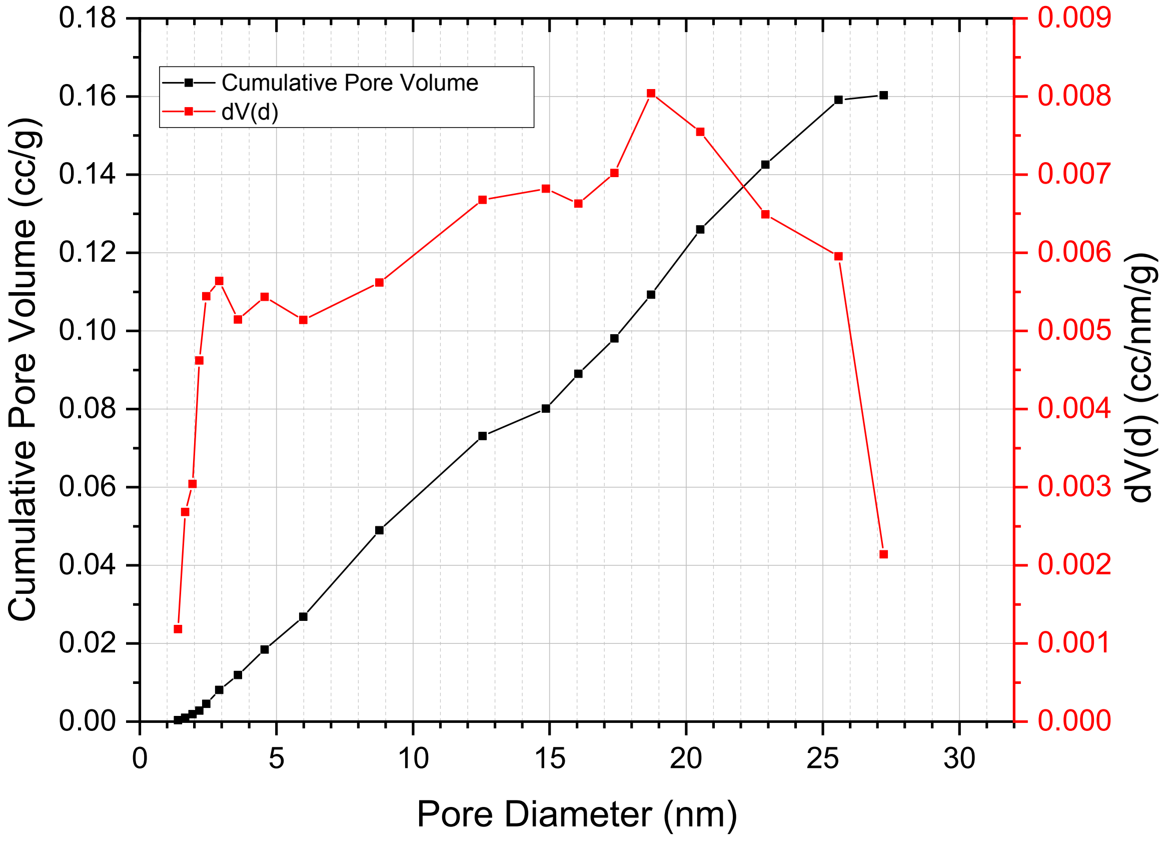

Figure 17: BJH pore size distribution of the BNNS-$MnFe_2O_4$ nanocomposite.

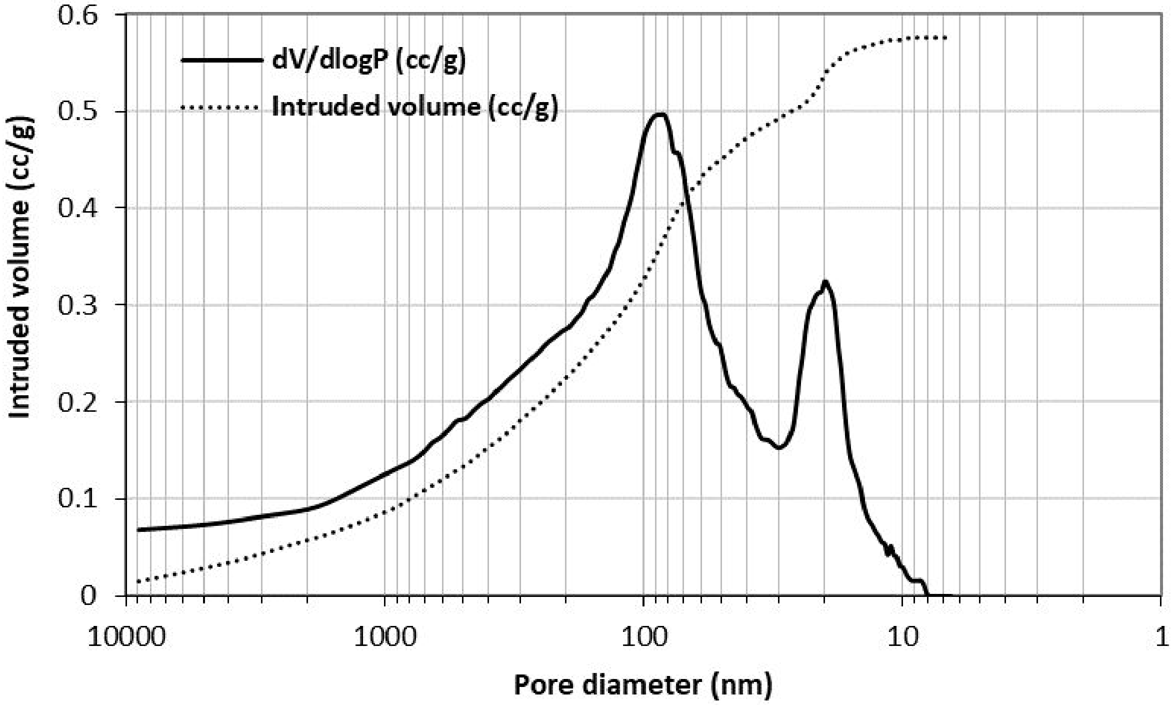

Figure S18: Mercury porosimetry intruded volume and pore size distribution (dV/dlogP) for BNNS.

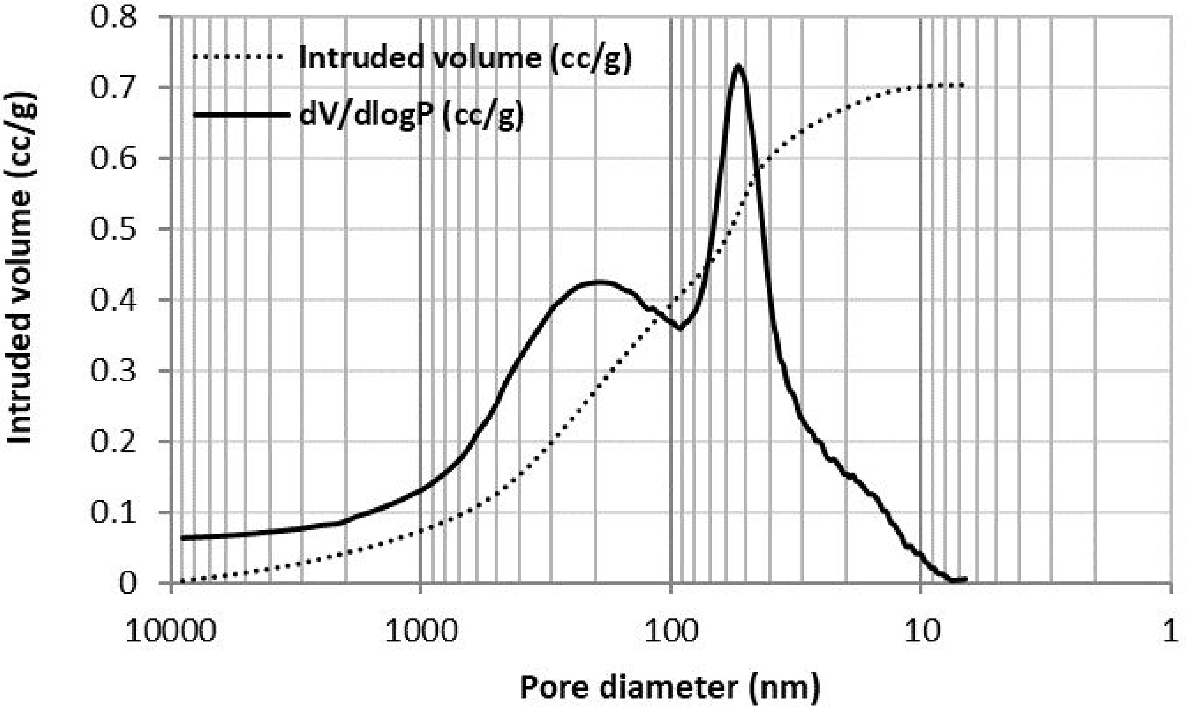

Figure S19: Mercury porosimetry intruded volume and pore size distribution (dV/dlogP) for BNNS-$MnFe_2O_4$.

Table S3: Flow Rates for BNNS and BNNS-$MnFe_2O_4$ membranes.

	BNNS ($Lm^{-2}hr^{-1}$)			BNNS-$MnFe_2O_4$ ($Lm^{-2}hr^{-1}$)		
	M1	M2	M3	M1	M2	M3
Run 1	678	551	632	445	363	402
Run 2	674	549	633	443	364	403
Run 3	675	548	635	444	365	403
Run 4	676	548	635	444	365	404
Run 5	679	550	634	445	362	405
Average	676	549	634	444	364	403
	**Average**		**620 ± 53**	**Average**		**404 ± 33**

Table S4: Physical characteristics for a membrane of 0.001018 m^2 operating at one bar.

	**BNNS**	**BNNS-$MnFe_2O_4$**
Mass	40 mg	40 mg
Thickness	0.050 mm	0.053 mm
Flow rate	620 $Lm^{-2}hr^{-1}$	404 $Lm^{-2}hr^{-1}$

Table S5: Percentage removal of the MB from 20 ml aliquots for the membranes and PVDF support using the 664 nm peak for analysis

**Cumulative MB volume (ml)**	**BNNS**	**BNNS-$CoFe_2O_4$**
	% removed	% removed
20	>99.9	>99.9
40	>99.9	>99.9
60	99.4	>99.9
80	98.8	99.0
100	98.1	98.7
120	77.7	89.0
140	50.0	72.5
160	16.67	45.0
180	4.1	28.5
200	0.1	12.0
220	0.0	1.1
240		0
260		0

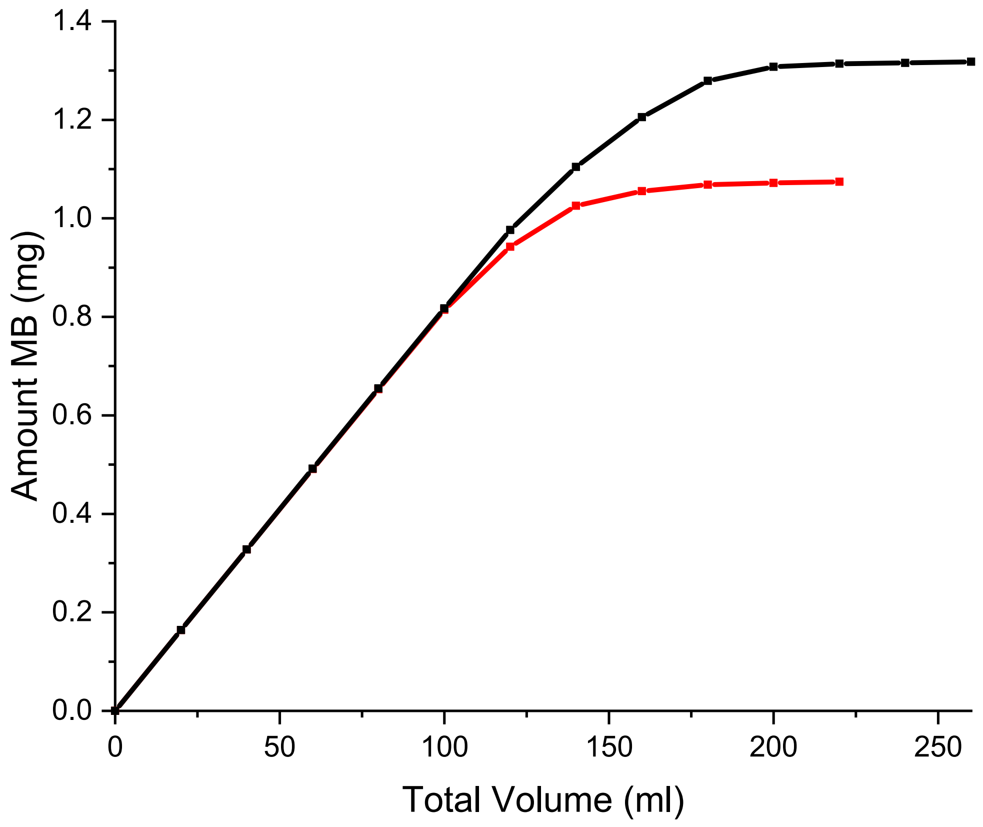

Figure S20: Accumulation of MB on BNNS (red line) and BNNS-$MnFe_2O_4$ (black line) membranes until saturation develops.

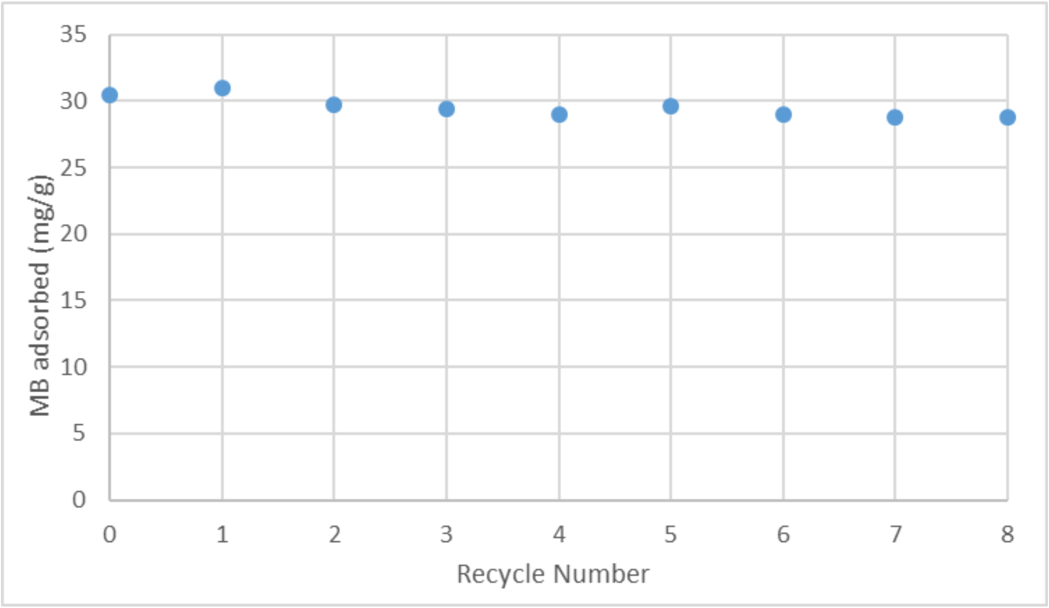

Figure S21: Recycling results for the BNN-$MnFe_2O_4$ nanocomposite

Table S6: Quantification of the MB removed with each recycle, showing the reduction in mass of the membrane material as some is lost in the recycling process.

Recycle Number	BNNS-$CoFe_2O_4$ mass (mg)	Adsorption (mg/g
0	41.1	30.5
1	40.2	31.0
2	39.1	29.7
3	37.9	29.4
4	36.3	29.0
5	36.1	29.6
6	35.8	29.0
7	35.4	28.8
8	35	28.8

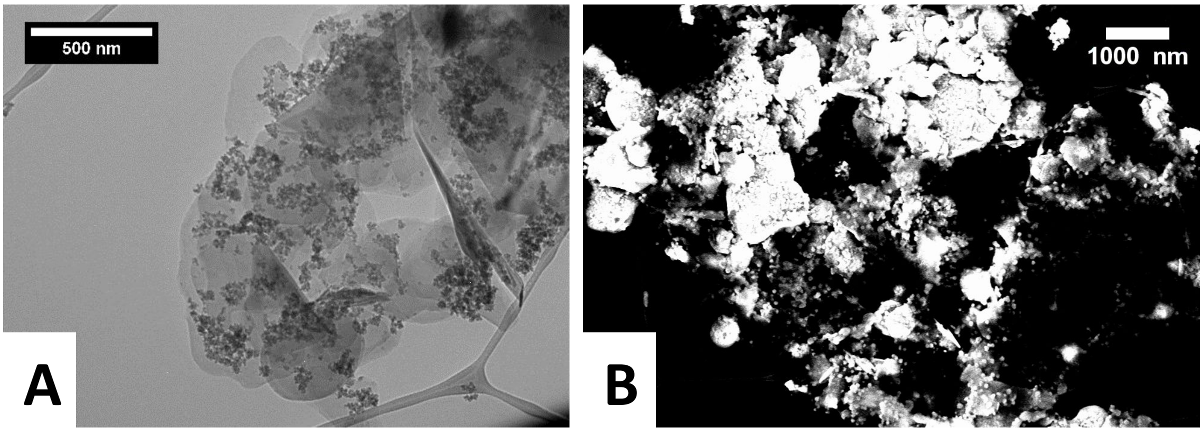

Figure S22: (A) TEM and (B) SEM images of the BNNS-$MnFe_2O_4$ nanocomposite after 8 recycles.

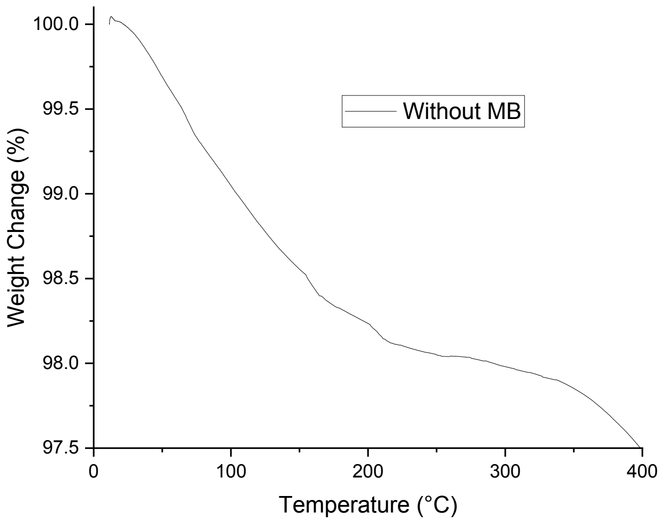

Figure S23: TGA of BNNS-$MnFe_2O_4$ without MB adsorbed.

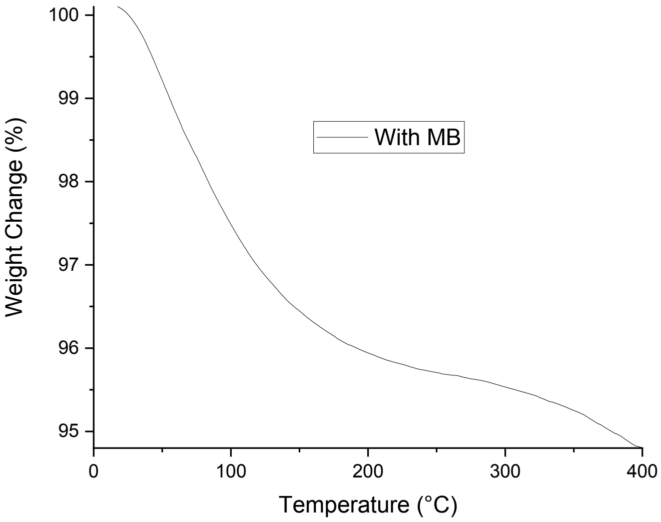

Figure S24: TGA of BNNS-$MnFe_2O_4$ with MB adsorbed.
